# The Sialomucin CD43 Drives Tuberculosis Immunopathology Across Different Host Genetic Backgrounds

**DOI:** 10.1007/s10753-026-02520-8

**Published:** 2026-06-10

**Authors:** Estefanía Alemán-Navarro, Dulce A. Mata-Espinosa, Manuel O. López-Torres, Jorge A. Barrios-Payán, Ángel F. Flores-Alcantar, Erika I. Melchy-Pérez, Rogelio Hernández-Pando, Yvonne Rosenstein

**Affiliations:** 1 Instituto de Biotecnología, Universidad Nacional Autónoma de México (UNAM), Campus Morelos, Cuernavaca, México; 2 Posgrado en Ciencias Bioquímicas, Universidad Nacional Autónoma de México (UNAM), Ciudad de México, México; 3 Instituto Nacional de Ciencias Médicas y Nutrición Salvador Zubirán, Ciudad de México, México

**Keywords:** *Mycobacterium tuberculosis*, CD43, Tuberculosis, Inflammatory profile, Cytokines, Mice

## Abstract

Tuberculosis (TB) remains a major global health challenge, with 10 million cases and 1.5 million deaths annually. Understanding the interplay between *Mycobacterium tuberculosis* (Mtb) and the host immune response is essential to better understand TB pathogenesis and develop new therapeutic strategies. Although the host immune bias strongly influences this interaction and shapes the ensuing response, the roles of specific immune receptors remain incompletely understood. Here, we investigated the role of CD43 in shaping the immune microenvironment during Mtb infection using Th2-biased BALB/c and Th1-biased C57BL/6 mice. CD43 deficiency consistently reduced pulmonary inflammation and improved survival in both strains, but through distinct mechanisms. In BALB/c mice, it enhanced early pulmonary cytokine production and promoted a more anti-inflammatory environment during late TB, leading to better control of bacterial growth and limiting the development of pneumonia. In contrast, in C57BL/6 mice, CD43 deficiency delayed pro-inflammatory cytokine responses and reduced the accumulation of inflammatory cells in the lungs, but it did not improve bacterial clearance. Together, these findings indicate that CD43 drives detrimental inflammation in TB through mechanisms that depend on the host immune bias, and highlight the complex and nuanced role of CD43 in regulating immune cell dynamics during TB progression, underscoring the need for tailored therapeutic approaches considering the context-dependent contributions of specific molecules such as CD43.

**Supplementary Information:**

The online version contains supplementary material available at 10.1007/s10753-026-02520-8.

## Introduction

Tuberculosis (TB) has re-emerged as the leading cause of death worldwide due to a single infectious agent after being surpassed by COVID-19 in 2023 [1]. Caused by the intracellular bacterium *Mycobacterium tuberculosis* (Mtb), TB primarily affects the lungs and poses major challenges for control. The licensed BCG vaccine, while preventing severe childhood disease, does not prevent pulmonary infection in children and adults, and treatment is hampered by lengthy regimens, severe side effects, and multidrug-resistant strains [2–4].

The host immune response to Mtb involves a delicate balance between protective immunity and pathological inflammation. Innate resident immune cells initiate defense by releasing pro-inflammatory mediators and recruiting inflammatory cells to the lungs. The adaptive immune response that follows promotes the generation, activation, and expansion of effector cells, further enhancing the recruitment of inflammatory cells, increasing their microbicidal activity, and limiting mycobacterial proliferation [5–7]. A subsequent anti-inflammatory response is necessary to mitigate the inflammation-associated damage during the progressive phase of the infection [8]. Key immune cells play dual roles in this inflammatory cascade: macrophages serve as both first-line defenders and bacterial reservoirs [9, 10]; neutrophils provide early protection but later exacerbate bacterial burden and tissue damage [11–14]; and CD4 and CD8 T cells modulate this balance by orchestrating immune cell recruitment and infected cell clearance [15–17]. The resulting immune microenvironment is shaped by the complex interplay of cytokines, chemokines, and cell subsets, ultimately determining the outcome, in which an optimal response restricts mycobacterial burden while preventing excessive host tissue damage [18].

The early interaction between Mtb and the immune system involves the recognition of mycobacterial pathogen-associated molecular patterns (PAMPs) by multiple receptors on immune cells. Beyond the well-characterized Toll-like receptors (TLRs), C-type lectin receptors (CLRs), and NOD-like receptors (NLRs), increasing attention has focused on mucins as immune regulators through their signaling capabilities [19, 20]. Among mucins, CD43, a transmembrane sialoglycoprotein expressed on all hematopoietic cells, including T cells, neutrophils, macrophages, and dendritic cells, except mature erythrocytes and resting B cells, has emerged as a relevant molecule in several infection contexts [21–34]. Because of its extended, rigid structure and its capacity to signal via its intracellular domain, CD43 has been hypothesized to be one of the first receptors to sense the extracellular microenvironment and direct the resulting cellular response by regulating processes that influence infection pathogenesis, such as cell adhesion, migration, activation, proliferation, survival, and cytokine and chemokine secretion [35–37].

Specific single-nucleotide polymorphisms (SNPs) in the CD43 gene have been linked to increased susceptibility to tuberculous meningitis, more severe disease, and reduced patient survival rates [38]. In macrophages, the primary cellular target of Mtb, CD43 recognizes the mycobacterial surface chaperones Cpn60.2 and DnaK, which are essential for Mtb viability and growth [39, 40], thereby facilitating macrophage-mycobacteria interaction [28, 41]. Besides showing reduced macrophage-Mtb binding, mice lacking CD43 display an increased pulmonary and splenic bacterial load during early and late-stage TB, and exacerbated granulomatous inflammation [25]. Their macrophages also produce less of the pro-inflammatory cytokines TNF-α, IL-6, and IL-12p40 due to low activation of the transcription factors NF-κB and AP-1 [26, 30].

However, these studies have primarily focused on the pro-inflammatory-biased C57BL/6 mouse model [25, 26, 28, 30], leaving a gap in understanding CD43’s contribution to TB pathogenesis in different immune contexts. Moreover, the role of CD43 in shaping the immune microenvironment at the different stages of TB remains poorly understood. Since Mtb-triggered inflammatory immune response significantly affects TB outcome [42, 43], and environmental factors in TB-endemic regions often promote an anti-inflammatory profile [44], understanding how CD43 influences disease progression in diverse immunological settings is critical. To address this gap, we explored the function of CD43 in TB in vivo on both Th2-biased (BALB/c) and Th1-biased (C57BL/6) mice, characterizing its impact on bacterial control, pulmonary and circulating cytokine production, immune cell infiltration, granuloma features, tissue damage, and survival, providing new insights into CD43’s host-dependent role in TB progression.

## Materials and Methods

### *Mycobacterium tuberculosis* H37Rv culture

Mtb virulent reference strain H37Rv (originally donated by Dr. Peter Small from Stanford University) was cultured in Middlebrook 7H9 broth (BD Biosciences) supplemented with oleic acid-albumin-dextrose-catalase (OADC, BD Biosciences), 0.5% glycerol, and 0.05% tyloxapol. Mycobacteria were grown at 35 °C with gentle agitation (70 rpm) until O.D. 600_nm_=1.348 (mid-log phase). The bacterial cultures were then centrifuged (10 min at 3000 rpm, 4 °C) and washed three times with sterile PBS. Bacterial pellets were resuspended in PBS, aliquoted, and stored at −70 °C. After thawing, sonication, and culturing in Middlebrook 7H10 agar (BD Biosciences), Colony Forming Units (CFUs) were counted to determine viable mycobacteria [45, 46].

### Mice

Male wild-type CD43^+/+^ (WT) and CD43^−/−^ (CD43KO) 8–10-week-old BALB/c and C57BL/6 mice were obtained from the SPF animal facility of the Instituto de Biotecnología of the Universidad Nacional Autónoma de México (IBT-UNAM). Male mice were selected for their well-characterized susceptibility to Mtb infection and accelerated disease pathology [47, 48], a model that facilitates the study of disease progression while controlling for sex-based variability. Mice were housed in groups of five in a ventilated rack with food and water *ad libitum*, with a 12:12 h light-dark cycle, and regulated temperature (23 ± 1 °C) and humidity (20–50%), at the Instituto Nacional de Ciencias Médicas y Nutrición Salvador Zubirán (INCMNSZ) P-3 biosecurity level animal facility. All experiments were performed according to the ARRIVE standards and Mexican norm NOM 062–Z00-1999, under the approval of the INCMNSZ’s Ethics Committee for Animal Experimentation (protocol number PAT-1983-19-21-1) and of the IBT-UNAM’s Ethics Committee (project 382).

### Genotypification and Phenotypification of BALB/c and C57BL/6 Mice

The genotype of each BALB/c and C57BL/6 WT and CD43KO mouse was verified through PCR. For each mouse, genomic DNA was isolated from the tail tip by using alkaline lysis. PCR was performed according to Jackson Laboratory recommendations, and the PCR products were visualized on a 1% agarose gel stained with GelRed (Biotium) (Fig. S1). For WT mice, the band size was 338 bp, and 200 bp for CD43KO mice, in which the CD43 gene is interrupted by a neomycin resistance (Neo) cassette [49].

At the time of sacrifice, the genotype of each mouse was confirmed by flow cytometry (Fig. S1). Briefly, 1 × 10^6^ thymus cells were centrifuged, fixed in 1.5% paraformaldehyde (PFA) for 10 min at 37 °C, washed, and resuspended in PBS. Next, cells were incubated for 30 min at 4 °C in 50 µL of FACS solution (PBS, 2% calf serum) supplemented with 20% mouse serum to block Fc receptors. Cells were then stained for 30 min at 4 °C with 1 µg/mL of anti-murine CD43-PE (clone S11, Biolegend), washed with 2 mL of FACS solution, centrifuged, and fixed with 2% PFA. An isotype-PE-stained control (1 µg/mL rat IgG2a, κ isotype, clone RTK2758, Biolegend) was included for each sample. Samples were acquired with a BD FACSCanto II flow cytometer using the BD FACSDiva Software v9.0.1, and analysis was performed with the BD FlowJo Software v10.

### Mice Infection, *post-mortem* Evaluation

WT and CD43KO BALB/c and C57BL/6 mice were anesthetized with sevoflurane (100 µL/mouse) in a 20 cm^3^ gas chamber and infected intratracheally (i.t.) with 2.5 × 10^5^ (BALB/c) or 8 × 10^3^ (C57BL/6) CFUs of *M. tuberculosis* in 100 µL of PBS. The inoculum for BALB/c mice was based on the progressive TB model established by Hernández-Pando et al. [50, 51]. For C57BL/6 mice, the dose was adjusted based on prior observations to induce active disease within a timeframe similar to that of the BALB/c model, while ensuring animal survival, given their pro-inflammatory response (data not published). WT and CD43KO mice were infected on the same day, in a blinded manner, with the same bacterial suspension to prevent bias or dosing errors. The concentration and viability of the inoculum were confirmed by CFU counts after plating serial dilutions on Middlebrook 7H10 agar. For BALB/c mice, each group included 5 WT and 5 CD43KO mice on post-infection days 3, 30, and 90, and 10 WT and 10 CD43KO mice on post-infection days 21, 60, and 120, as these time points are critical for infection progression [50]. For C57BL/6 mice, a single experiment was conducted with groups of 5 WT and 5 CD43KO mice at days 30, 60, and 120.

At the indicated times, mice were euthanized by exsanguination under intraperitoneal anesthesia (sodium pentobarbital, 210 mg/kg). Blood was collected, and serum was extracted and stored at − 70 °C for cytokine measurement. To assess the lung cytokine profile, the right lung was aseptically removed, weighed, cut into small pieces, and incubated for 8 h (37 °C, 5% CO_2_) in a microtube with 1 mL of RPMI culture medium (HyClone) supplemented with 2 mM glutamine. The samples were then centrifuged (10 min, 10,000 rpm), and the supernatant was transferred to another microtube, further centrifuged (10 min, 14,000 rpm) to remove cellular debris, and stored at − 70 °C. The left lung was fixed for histopathological analysis by perfusion through the trachea with absolute ethanol.

### Pulmonary Bacterial Load Count

The bacterial load in each lung was measured by CFU count [45]. Briefly, the right lung fragments were homogenized in 1 mL of PBS containing 0.05% Tween 80 using the FastPrep system (MP Biomedicals). Samples were sonicated and vortexed to break up bacterial aggregates, and four serial dilutions (10^− 1^ to 10^− 4^) of the homogenized tissue were plated in duplicate onto Middlebrook 7H10 agar supplemented with OADC. The plates were incubated for 21 days at 37 °C and 5% CO_2_, and CFUs were counted with a magnifying glass. Bacterial loads were normalized per gram of lung tissue to account for differences in organ size.

### Cytokine Profile

Cytokines were quantified in lung culture supernatants and serum samples with the LEGENDplex Mouse Inflammation Panel 13-plex (BioLegend) (pro-inflammatory: IFN-γ, TNF-α, IL-12p70, IL-1α, IL-1β, IL-6, GM-CSF, MCP-1; Th17-related: IL-23, IL-17A; and anti-inflammatory: IL-10, IL-27, IFN-β) following the manufacturer’s instructions. Samples were acquired with a BD FACSCanto II flow cytometer with the BD FACSDiva software v9.0.1, and the analysis was performed with the LEGENDplex Data Analysis Software (BioLegend, https://legendplex.qognit.com*).* The anti-inflammatory cytokine IL-4 was quantified by ELISA with a Mouse ELISA Ready-SET-Go! Kit (eBiosciences), following the manufacturer’s instructions. The pulmonary cytokine concentration for each sample was expressed as pg/mL per gram of lung. To facilitate comparison across groups and time points on a common scale, cytokine data were normalized to z-scores using the pooled mean and standard deviation of all WT and CD43KO samples as reference, resulting in a zero-centered distribution, with positive and negative values indicating levels above or below the global mean. Hierarchical clustering of cytokine z-scores was then performed using Euclidean distance and the average linkage method with MeV software v4.8. Statistical analysis was performed using the two-tailed Mann-Whitney U test with a 95% confidence interval, in GraphPad Prism Software v6.

### Histologic and Morphometric Analysis of the Lungs

Lungs fixed with absolute ethanol were cut parasagittally, sequentially dehydrated, cleared, and infiltrated with paraffin using the STP-120 system (Thermo Scientific). Processed lungs were embedded in paraffin, and 4 μm-thick tissue slices were obtained and stained with Hematoxylin and Eosin (H&E). The histopathological analysis concentrated on inflammatory infiltrates, pneumonia, and granulomas. Inflammatory infiltrates involve focal accumulations of immune cells in specific pulmonary structures that do not fill the alveolar lumen or cause consolidation. Pneumonia is characterized by abundant inflammatory cells filling the alveolar lumen, resulting in consolidation. In mice, TB granulomas are typically characterized by organized, compact aggregates of immune cells, such as macrophages and lymphocytes, and unlike human TB granulomas, usually lack significant necrosis.

We evaluated alveolar-capillary interstitial (INT), perivascular (PV), and peribronchial (PB) inflammatory infiltrates across the upper, middle, and lower lung regions. Specifically, 27 measurements of INT infiltrate area (9 per section), and nine measurements of PV or PB infiltrate area (3 per section) were obtained. PV infiltrates were measured around 100–150 μm diameter blood vessels, and PB infiltrates around 200–250 μm-diameter bronchi. The total granuloma area was calculated by counting and measuring all granulomas in the entire lung. The extent of pneumonia was evaluated by measuring the total lung area and the percentage of the lung affected by pneumonia. Images were captured with a Leica Q500 microscope (20X for INT and PV, 10X for PB, 40X for granulomas, and 20X for pneumonia regions) with the Image Analysis System (Milton Keynes, UK) and analyzed with the Leica Application Suite Software v4.3. Statistical analysis was performed with the GraphPad Prism Software v6, using the two-tailed Mann-Whitney U test with a 95% confidence interval.

### Pulmonary Immune Cell Quantification Analysis

For immunohistochemistry (IHC), 4 μm-thick sections were cut from paraffin-embedded lungs and mounted on silane-coated slides (Biocare). Samples were deparaffinized in xylene and rehydrated by sequential incubations on 50% xylene-50% ethanol, absolute ethanol, 96% ethanol, 80% ethanol, and distilled water. For antigen retrieval, slides were treated for 20 min at 60 °C with Liberate Antibody Binding Solution (Polysciences). After washing with PBS-0.05% Tween 20 (PBS-T), endogenous peroxidase was blocked with a 3% H_2_O_2_ solution for 30 min with agitation (50 rpm). To prevent nonspecific antibody binding, slides were then washed with PBS-T and incubated with blocking solution (5% human serum, 5% horse serum, 1% BSA, and 0.2% casein in PBS) for 2 h with agitation. To assess pulmonary macrophages (F4/80+), neutrophils (Ly6G+), helper T lymphocytes (CD4+), and cytotoxic T lymphocytes (CD8+), sections were incubated overnight in a humidity chamber with agitation with biotin-labeled anti-mouse F4/80 (10 µg/mL, clone BM8, Biolegend), Ly6G (6.6 µg/mL, clone 1A8, Biolegend), CD4 (3.3 µg/mL, clone 4SM95, eBioscience) or CD8 (3.3 µg/mL, clone 4SM15, eBioscience), diluted in blocking solution. For detection, slides were washed with PBS-T, incubated with Mouse Immunodetector HRP (BioSB) for 10 min at room temperature with agitation, washed, incubated with DAB Chromogen (1:100 dilution) in Polydetector DAB Buffer Solution (BioSB) for 10 min, and washed with distilled water. Nuclei were counterstained with hematoxylin, and samples were immersed in an aqueous saturated lithium carbonate solution (1.3%) for bluing. Finally, samples were dehydrated by sequential incubations in 96% ethanol, absolute ethanol, 50% ethanol-50% xylene, and xylene, and then mounted in Entellan medium (Sigma-Aldrich) for microscopy analysis. A negative control (no primary antibody) was included (Figs. S6 and S10).

Images of inflammatory infiltrate (INT, 20X; PV, 10X/20X; PB, 10X/20X), alveolitis or pneumonia regions (20X), and granulomas (63X) were acquired with an Olympus IX81F-3 microscope coupled to a ZEISS AxioCam 208 color camera, using the Zen (Blue Edition) Software v3.1. F4/80+, Ly6G+, CD4+, and CD8 + cells per inflammatory region were quantified in 10 fields across the upper, middle, and lower lung areas using the QuPath 0.5.1 software [52], with manual definition of regions of interest, and optimized detection parameters to ensure an accurate quantification of positive cells per mm^2^. Statistical analysis was performed with the GraphPad Prism Software v6, using the two-tailed Mann-Whitney U test with a 95% confidence interval.

## Results

### CD43 deficiency improves control of Mtb infection and increases survival in the late stage of TB in BALB/c mice

**Fig. 1:**
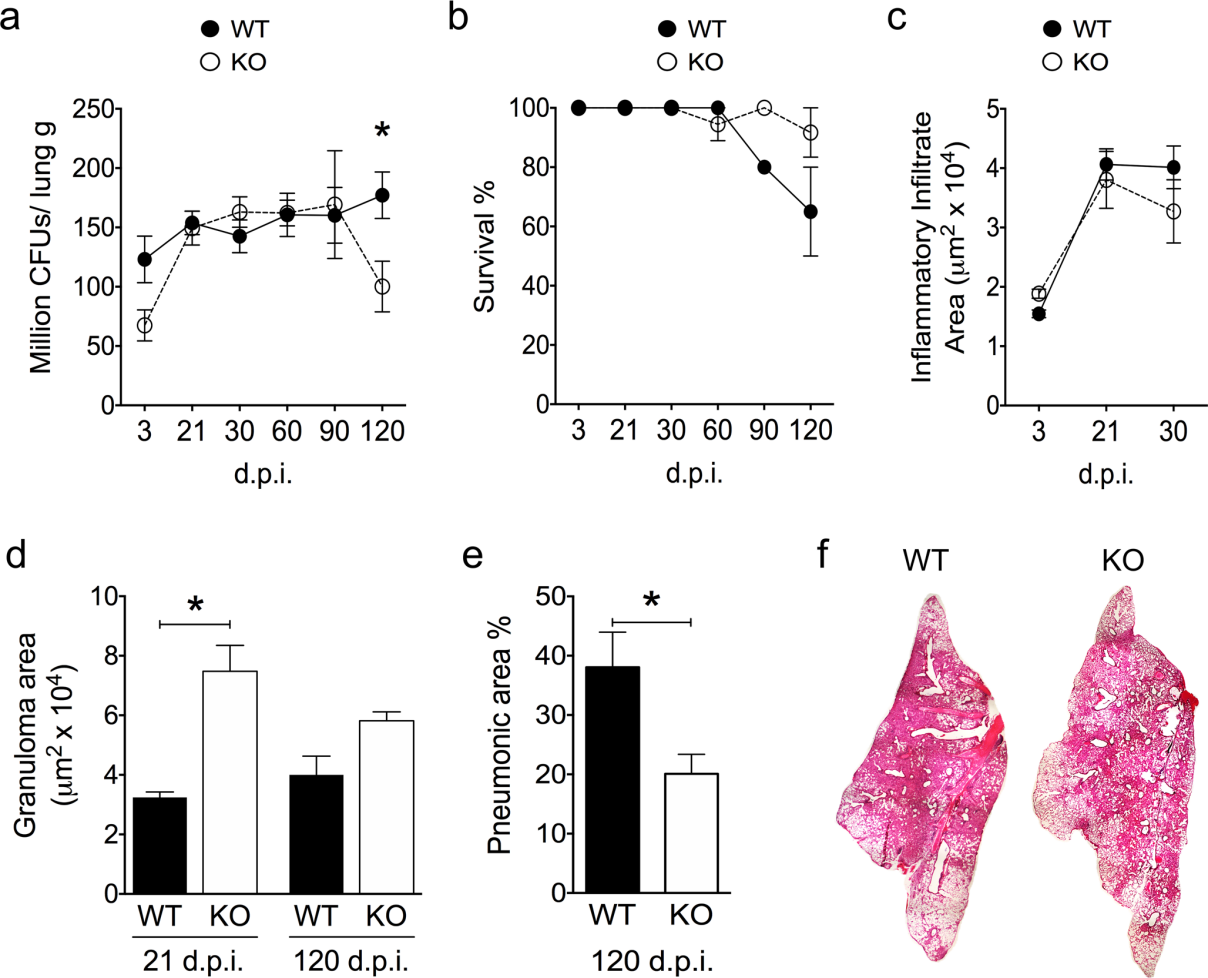
CD43 deficiency enhances early granuloma formation and improves the outcome in BALB/c mice with TB. (**a**) Pulmonary bacterial load in WT and CD43KO mice at 3, 21, 30, 60, 90, and 120 days post-infection (d.p.i.), measured by CFU count in lung homogenates and normalized to each mouse’s organ weight. (**b**) Survival percentage of mice at the time points shown in a. (**c**) Area of total cellular inflammatory infiltrate in the lungs during early TB (3, 21, and 30 d.p.i.); morphometric analysis. (**d**) Total pulmonary granulomatous area at 21 and 120 d.p.i.; morphometric analysis. (**e**) Percentage of pneumonic area relative to total lung area at 120 d.p.i.; morphometric analysis. (**f**) Representative H&E-stained lung sections at 120 d.p.i. *n* = 5 mice/group (days 3, 30, and 90) and *n* = 10 mice/group (days 21, 60, and 120). Statistical analysis: two-tailed Mann-Whitney U test; *p < 0.05. Error bars: SEM

Due to their Th2-dominant response, BALB/c mice can tolerate high infective doses of Mtb and develop severe TB, with an early phase extending one month after infection, followed by a progressive or late phase [50]. Consistent with reports showing that C57BL/6 CD43-deficient macrophages exhibit reduced Mtb binding and a lower initial intracellular burden that subsequently matches WT levels due to faster bacterial growth [22, 25, 26, 28], BALB/c CD43KO mice also showed a lower pulmonary bacterial load at 3 days post-infection (d.p.i.), with a 50% reduction on average compared to their WT counterparts, matching that of WT mice by 21 d.p.i. Remarkably, by 120 d.p.i., CD43KO mice had an average lung mycobacterial burden that was 40% lower and a higher survival rate (Figs. 1a and b).

In the BALB/c TB model, the first month post-infection is characterized by abundant inflammatory infiltrate in the alveolar-capillary, perivenular, and peribronchial regions of the lungs, as well as granuloma formation [50]. Lung histopathology revealed no significant differences in the extent of inflammatory infiltrate between WT and CD43KO mice during this period (Figs. 1c and S2a). At 21 d.p.i., however, when granulomas mature, the lungs of CD43KO mice showed a larger granulomatous area due to larger individual granulomas (Figs. 1d and S2b). In WT mice, the granuloma size increased as the infection progressed, matching that of CD43KO mice by 120 d.p.i. (Fig. S2b). The number of granulomas was not significantly affected by CD43 deficiency and decreased over time in both groups (Fig. S2c). Consistent with improved mycobacterial containment, by 120 d.p.i., the area covered by pneumonia, which progressively develops during late TB, was half the size in CD43KO mice compared to that of WT mice (Figs. 1e and f).

Thus, in BALB/c mice, CD43 deficiency favors larger granulomas during the early phase of TB, and results in better control of the pulmonary mycobacterial load, less tissue damage during the later stage of the disease, and enhanced survival rates.

### CD43 Deficiency Modifies the Cytokine Landscape in the Lungs of Mtb-Infected BALB/c Mice and Attenuates Late-Stage Inflammation

In BALB/c mice, early TB is marked by an initial pro-inflammatory response that peaks at 21 d.p.i [50]. We evaluated pro-inflammatory cytokines (IL-1α, IL-1β, IL-12p70, IFN-γ, TNF-α, IL-6, GM-CSF, and MCP-1), which drive inflammation and limit Mtb survival; anti-inflammatory cytokines (IL-4, IL-10, IL-27, IFN-β), which may facilitate disease progression but also mitigate the inflammation-associated tissue damage, and Th17-related cytokines (IL-23, IL-17A), which recruit immune cells to the lungs and help restrict bacterial growth [53–55]. In the lungs of both WT and CD43KO mice, levels of IL-12p70, IFN-γ, TNF-α, IL-1α, IL-1β, MCP-1, IL-17A, and IL-10 increased during early TB, while the concentration of IL-23, IL-27, and IL-4 declined sharply after 3 d.p.i. and remained stable thereafter (Fig. S3).

**Fig. 2:**
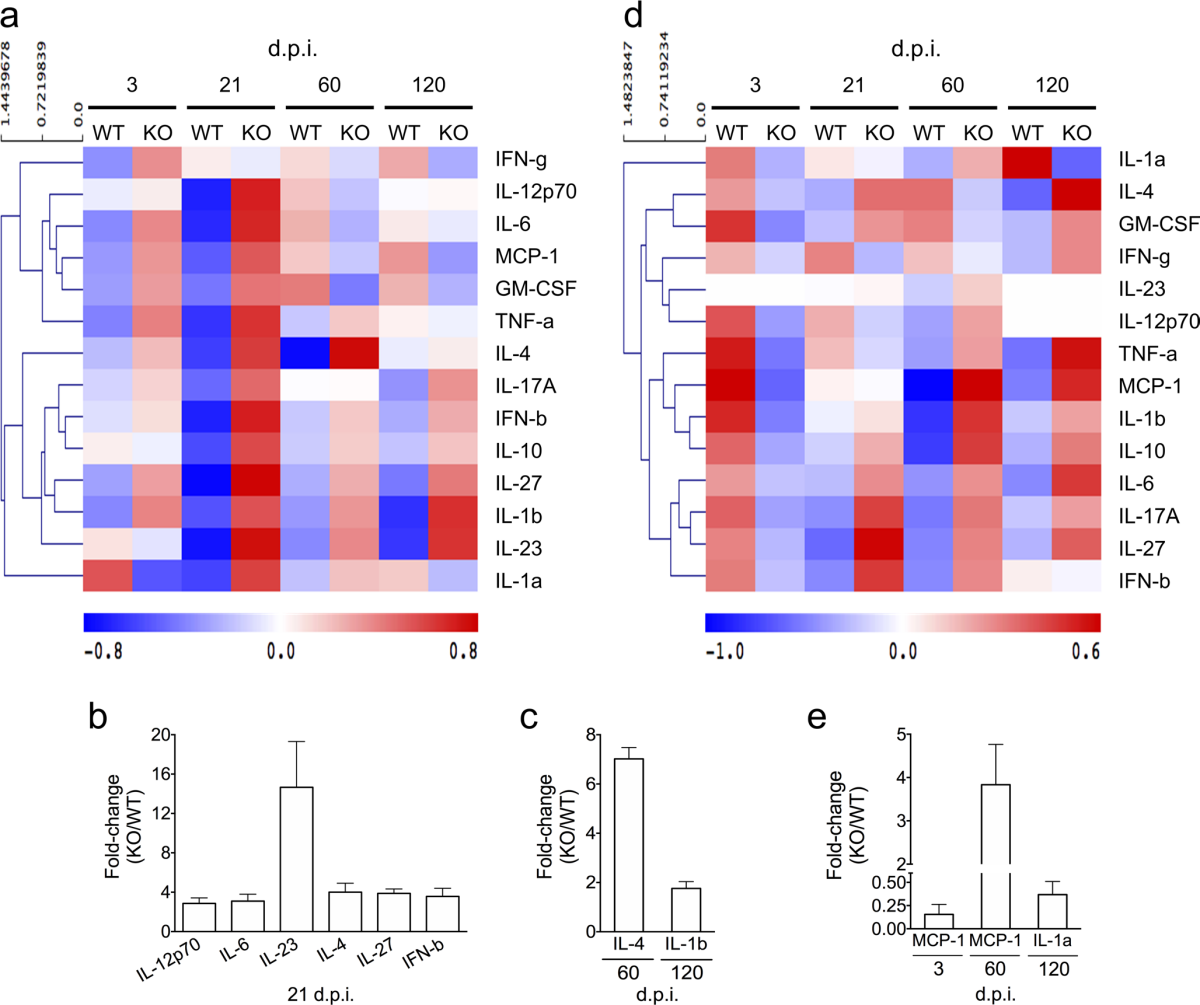
CD43 deficiency shifts the pulmonary cytokine environment in BALB/c mice toward an anti-inflammatory profile in late-stage TB. (**a**) Hierarchically clustered Z-scores of pulmonary pro-inflammatory (IL-1α, IL-1β, IL-12p70, IFN-γ, TNF-α, IL-6, GM-CSF, MCP-1), Th17-related (IL-23, IL-17A), and anti-inflammatory cytokines (IL-4, IL-10, IL-27, IFN-β) in WT and CD43KO mice at 3, 21, 60, and 120 d.p.i. (**b**) Ratio of pulmonary cytokine concentrations (CD43KO/WT) with significant differential expression between groups at 21 d.p.i., and at (**c**) 60 and 120 d.p.i. (**d**) Hierarchically clustered Z-scores of circulating cytokine production in WT and CD43KO mice at 3, 21, 60, and 120 d.p.i. (**e**) Ratio of circulating cytokine concentrations (CD43KO/WT) with significant differential expression between groups at 3, 60, and 120 d.p.i. *n* = 5 mice/group (day 3) and *n* = 10 mice/group (days 21, 60, and 120)

At 3 d.p.i., the CD43KO lungs showed higher levels of IFN-γ, IL-1β, TNF-α, IL-6, GM-CSF, MCP-1, and IL-27, while IL-1α levels were lower compared to WT controls. By 21 d.p.i., all cytokines except IFN-γ were increased in the CD43KO group (Fig. 2a), with marked increases in IL-12p70 (3.5-fold), IL-6 (3.5-fold), and IL-23 (14-fold), along with a 4-fold increase in the anti-inflammatory cytokines IL-4, IL-27, and IFN-β (Fig. 2b). In late-stage TB, CD43KO mice maintained higher levels of Th17-related and anti-inflammatory cytokines (Fig. 2a), with significantly elevated IL-4 (7-fold at 60 d.p.i.) and IL-1β (2-fold at 120 d.p.i.) (Fig. 2c). In contrast, most pro-inflammatory cytokines were reduced or unchanged during late infection in these mice (Fig. 2a).

Unlike the pulmonary profile, at 3 d.p.i., serum cytokine levels in CD43KO mice were lower than in WT mice, with MCP-1 showing a significant 4-fold reduction. By 21 d.p.i., however, IL-6, GM-CSF, IL-17A, IL-4, IL-10, IL-27, and IFN-β were increased in the CD43KO group. This increase persisted through 60 d.p.i. for IL-6, IL-17A, IL-10, IL-27, and IFN-β, and extended to IL-1α, IL-1β, IL-12p70, TNF-α, MCP-1, and IL-23, while GM-CSF and IL-4 levels decreased. At 60 d.p.i., MCP-1 had shifted to 4-fold higher than WT controls. By 120 d.p.i., CD43KO mice exhibited higher circulating levels of most cytokines than WT mice, except for IL-12p70, IL-23, and IFN-β, which remained unchanged, and IL-1α, which was 2-fold lower (Figs. 2d-e and S4).

Together, these data suggest that CD43 deficiency in BALB/c mice leads to increased pulmonary production of several pro-inflammatory, Th17-related, and anti-inflammatory cytokines during early TB, particularly at the peak of inflammation. The persistent upregulation of anti-inflammatory cytokines during late TB, coupled with the downregulation of many pro-inflammatory cytokines, likely contributes to reduced local inflammation-associated damage in the late phase of the disease, thereby improving survival.

### CD43 Deficiency Alters Pulmonary Myeloid and Lymphoid Cell Dynamics During Mtb Infection in BALB/c Mice

Pulmonary infiltration of macrophages, neutrophils, and T lymphocytes reflects a protective response, as these cells control bacterial burden and the inflammatory process. In addition to their participation in inflammatory infiltration, macrophages and T lymphocytes constitute the main components of granulomas. However, their accumulation can promote mycobacterial persistence and drive tissue damage by fostering inflammation and the development of pneumonia [50, 56]. Given the marked differences in cytokine profile (21 d.p.i.), pneumonia, and survival (120 d.p.i.) between WT and CD43KO mice, along with the role of CD43 in cell migration [57–62], we assessed whether CD43 deficiency altered the spatial distribution and abundance of pulmonary macrophages, neutrophils, and T lymphocytes (CD4 + and CD8+) at 21 and 120 d.p.i.

**Fig. 3:**
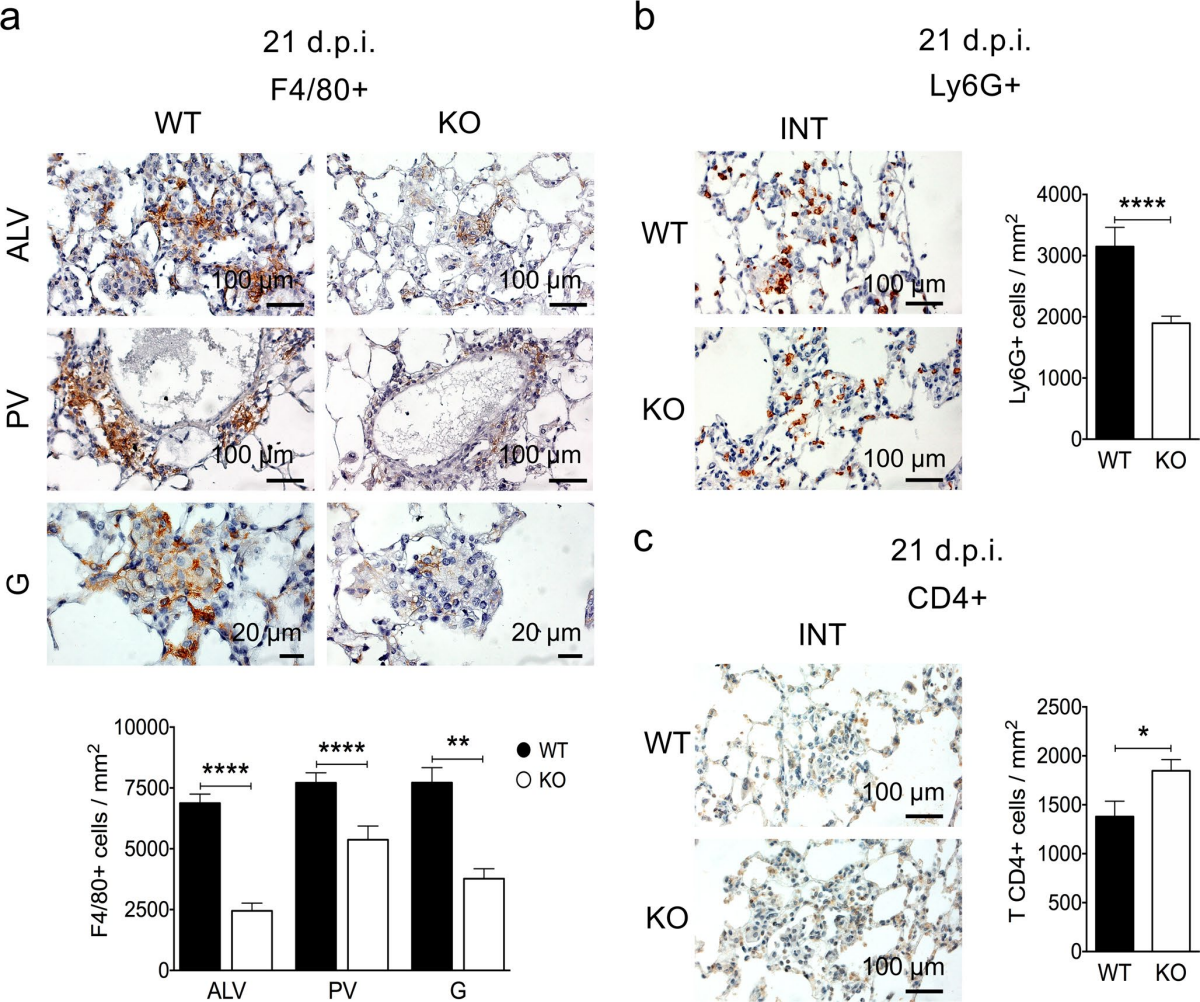
Early pulmonary macrophage, neutrophil, and CD4 T cell abundance is altered in CD43KO BALB/c mice, in a compartment-specific manner. Representative immunohistochemistry images show pulmonary immune cell populations (brown staining) in alveolar-capillary interstitial (INT, 20X) and perivascular (PV, 20X) inflammatory infiltrate, alveolar inflammation areas (ALV, 20X), and granulomas (G, 63X) at 21 d.p.i. Plots show the abundance (cells/mm²) of immune cell populations in manually annotated regions of lung sections. (**a**) Macrophages (F4/80+). (**b**) Neutrophils (Ly6G+). (**c**) CD4 + T cells. *n* = 10 mice/group. Statistical analysis: two-tailed Mann-Whitney U test; *p < 0.05, **p < 0.01, ****p < 0.0001. Error bars: SEM

Consistent with their critical role in the early response, at 21 d.p.i, macrophages were abundant in alveolar, perivascular, and peribronchial inflammatory infiltrates, as well as within granulomas. CD43KO mice showed a 1.5- to 3-fold decrease in macrophage numbers, except in the peribronchial regions, where macrophage numbers were not significantly different from those in WT mice (Figs. 3a and S5a). Neutrophils were predominantly abundant in the alveolar inflammatory infiltrate, with a 2-fold decrease in CD43KO mice (Fig. 3b). CD4 and CD8 T cells were distributed across alveolar, perivascular, and peribronchial inflammatory infiltrates, as well as granulomas. Notably, CD43KO mice harbored 30% more CD4 T cells in the alveolar regions than WT controls (Fig. 3c), while no significant differences were detected in CD8 T cell abundance across any compartment (Figs. S5b and c).

**Fig. 4:**
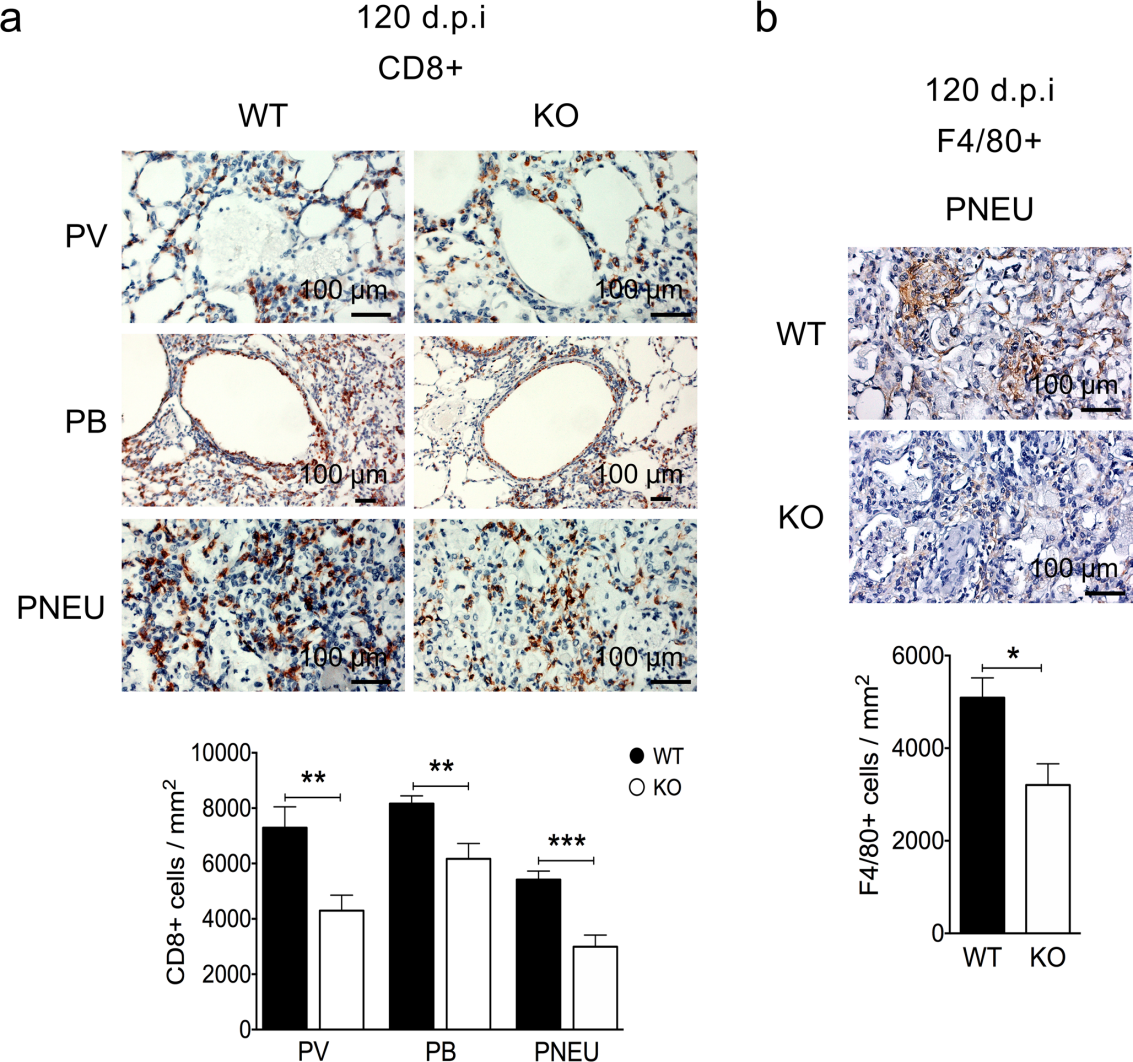
CD43 deficiency modifies pulmonary immune cell distribution in late-stage TB in BALB/c mice. Representative immunohistochemistry images of pulmonary immune cell populations (brown staining) in areas of pneumonia (PNEU, 20X), perivascular (PV, 20X), and peribronchial (PB, 10X) inflammatory infiltrate at 120 d.p.i. Plots show the abundance (cells/mm²) of immune cell populations, determined in manually annotated regions of lung sections. (**a**) CD8 + T cells in inflammatory infiltrate areas. (**b**) Macrophages (F4/80+) in pneumonic areas. *n* = 10 mice/group. Statistical analysis: two-tailed Mann-Whitney U test; *p < 0.05, **p < 0.01, ***p < 0.001. Error bars: SEM

During late TB (120 d.p.i.), macrophages were confined to the alveolar inflammatory infiltrate, while CD4 T cells localized to the perivascular areas and zones with dense cellular infiltrate scattered throughout the lungs, and were equally abundant in both WT and CD43KO mice (Figs. S5d and e). In contrast, CD8 T cells were less abundant in perivascular and peribronchial inflammatory regions of the CD43KO lungs (Fig. 4a). Pneumonic areas in both groups contained primarily macrophages, neutrophils, and CD8 T cells, with minimal infiltration of CD4 T cells (Figs. 4a, b, and S5f). CD43KO mice had fewer macrophages and CD8 T cells than WT mice, with most macrophages showing low or no F4/80 expression (Figs. 4a and b). In late granulomas, although the abundance of macrophages and T cells was similar between groups, both WT and CD43KO mice showed increased numbers of CD4 and CD8 T cells relative to early TB, with CD43KO mice additionally showing increased macrophage density (Fig. S5g).

Collectively, the CD43-regulated cell distribution and abundance of immune cell populations in infected BALB/c mice are consistent with the tissue pathology and disease outcomes. The early reduction in macrophages and neutrophils in CD43KO mice likely limits the initial amplification of inflammation, while the late-stage decrease in macrophages and CD8 T cells may contribute to attenuated pathology. These findings suggest that CD43 exacerbates inflammatory lung damage and mortality by promoting the recruitment of detrimental immune cells to the lungs.

### CD43 Deficiency Differentially Modulates Mtb Control but Improves Survival in Both C57BL/6 and BALB/c Mice

To assess whether the role of CD43 was strain-specific, C57BL/6 WT and CD43KO mice were infected with 8 × 10^3^ CFUs of Mtb H37Rv, a dose that drives progressive disease while avoiding lethal inflammatory damage associated with the C57BL/6 strain’s Th1-biased response. We evaluated disease progression at early (30 d.p.i.), intermediate (60 d.p.i.), and late-phase (120 d.p.i.) time points, mirroring the phases in which CD43-dependent differences emerged in BALB/c mice.

**Fig. 5:**
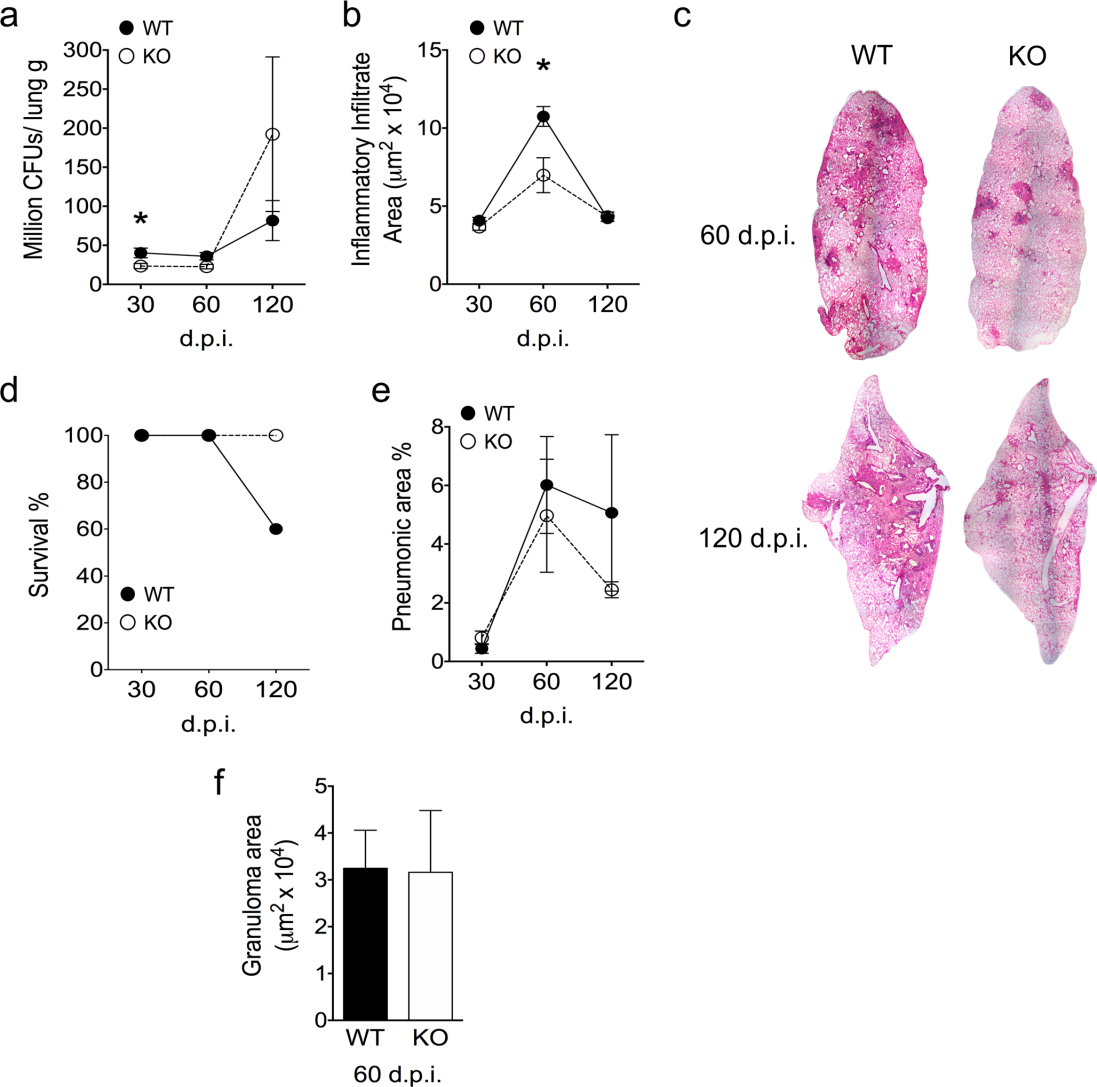
In C57BL/6 mice, CD43 deficiency leads to reduced immunopathology and increased survival despite failing to limit bacterial control during late TB. (**a**) Pulmonary bacterial load in WT and CD43KO mice at 30, 60, and 120 d.p.i., assessed by CFU count in lung homogenates and normalized to each mouse’s lung weight. (**b**) Total area of cellular inflammatory infiltrate in the lungs at 30, 60, and 120 d.p.i.; morphometric analysis. (**c**) Representative H&E-stained lung sections at 60 and 120 d.p.i. (**d**) Survival percentage of mice at 30, 60, and 120 d.p.i. (**e**) Percentage of pneumonic area relative to total lung area at 30, 60, and 120 d.p.i.; morphometric analysis. (**f**) Total pulmonary granulomatous area at 60 d.p.i.; morphometric analysis. *n* = 5 mice/group. Statistical analysis: two-tailed Mann-Whitney U test; *p < 0.05. Error bars: SEM

As expected [25, 26], C57BL/6 CD43KO mice had lower bacterial burden at 30 and 60 d.p.i. than WT mice. However, unlike BALB/c CD43KO mice, which caught up with the WT group by 21 d.p.i., the C57BL/6 CD43KO mice did not match the bacterial load of their WT counterparts until 120 d.p.i (Fig. 5a). Notably, despite receiving a 30-fold lower inoculum, C57BL/6 WT mice still achieved half the pulmonary load of BALB/c WT mice by 120 d.p.i,

The kinetics of the inflammatory infiltrate also differed between BALB/c and C57BL/6 mice. Unlike BALB/c mice, in which infiltration plateaued during early TB, C57BL/6 mice developed a more intense inflammatory infiltrate that peaked at 60 d.p.i. and then declined (Figs. 5b and S7a). Consistent with the notion that excessive cellular infiltration promotes mortality in TB [44], C57BL/6 WT mice showed a more extensive inflammatory infiltrate than CD43KO mice at the 60 d.p.i. peak (Figs. 5b and c), correlating with decreased survival (Fig. 5d). Notably, this difference in survival occurred despite a comparable pulmonary bacterial load in WT and CD43KO groups by 120 d.p.i. In contrast to the BALB/c TB model, independent of CD43 expression, C57BL/6 mice showed limited pneumonic regions (Fig. 5e), and the absence of CD43 did not impact the granuloma size (Fig. S7b), number (Fig. S7c), or total area occupied (Fig. 5f).

Collectively, these data suggest that CD43 deficiency impairs the control of the pulmonary Mtb load in both C57BL/6 and BALB/c strains. Remarkably, it improved survival rates during late TB in both strains through distinct mechanisms: by mitigating inflammatory cellular infiltration without altering bacterial load in the lungs of C57BL/6 mice, and by limiting bacterial burden and lung immunopathology in BALB/c mice.

### CD43 Deficiency Delays the Pro-Inflammatory Response in Mtb-Infected C57BL/6 Mice

**Fig. 6:**
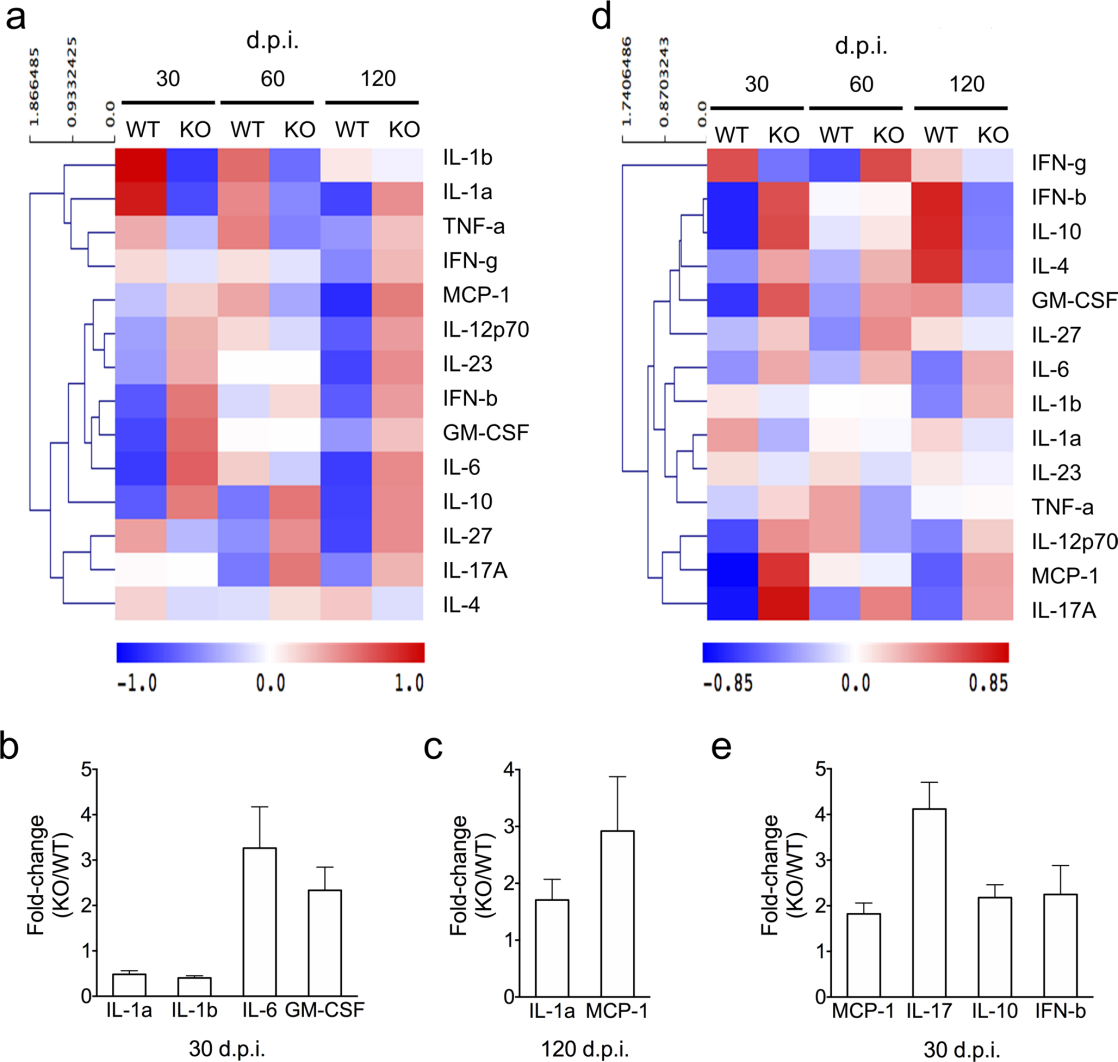
The absence of CD43 delays the inflammatory response in C57BL/6 mice during late TB. (**a**) Hierarchical clustering of Z-scores of pulmonary pro-inflammatory (IL-1α, IL-1β, IL-12p70, IFN-γ, TNF-α, IL-6, GM-CSF, MCP-1), Th17-related (IL-23, IL-17A), and anti-inflammatory (IL-4, IL-10, IL-27, IFN-β) cytokines in WT and CD43KO mice at 30, 60, and 120 d.p.i. (**b**) Ratio (CD43KO/WT) of pulmonary cytokine concentrations showing significant differences between groups at 30 d.p.i. and (**c**) 120 d.p.i. (**d**) Hierarchical clustering of Z-scores of circulating cytokine production in WT and CD43KO mice at 30, 60, and 120 d.p.i. (**e**) Ratio (CD43KO/WT) of circulating cytokine concentrations showing significant differences between groups at 30 d.p.i. *n* = 5 mice/group

To further characterize the inflammatory milieu in the C57BL/6 background, we assessed cytokine levels in the lungs of WT and CD43KO mice. In contrast to the broad increase in pulmonary cytokines observed in BALB/c CD43KO mice during early TB, C57BL/6 CD43KO mice exhibited specific changes at 30 d.p.i., with elevated IL-10 and IFN-β, significantly higher IL-6 (3-fold) and GM-CSF (2.5-fold), and significantly reduced levels of IL-1α and IL-1β (2-fold) compared to WT controls. By 60 d.p.i., the lungs of CD43-deficient C57BL/6 mice had higher IL-17A, IL-10, and IL-27 relative to the WT group, along with reduced levels of the pro-inflammatory IL-1α, IL-1β, TNF-α, and MCP-1. Surprisingly, by 120 d.p.i., C57BL/6 CD43KO mice had higher levels of most cytokines except IL-1β and IL-4, with significant increases in IL-1α (2-fold) and MCP-1 (3-fold) compared to WT mice (Fig. 6a-c and S8).

At 30 d.p.i., C57BL/6 CD43KO mice exhibited a systemic cytokine profile characterized by reduced levels of circulating IFN-γ, increased IL-12p70, IL-6, and GM-CSF, and significantly increased levels of MCP-1 (1.8-fold), IL-17A (4-fold), IL-10 (2-fold), and IFN-β (2-fold) compared to WT mice (Figs. 6d-e and S9). Consistent with the lung data, CD43-deficient mice exhibited a trend toward higher IL-17A, IL-27, and GM-CSF at 60 d.p.i. Notably, while CD43 deficiency induced a global increase in circulating cytokines in BALB/c mice by 120 d.p.i., the response in C57BL/6 CD43KO mice was more restricted as only IL-17A and specific pro-inflammatory cytokines remained elevated, whereas regulatory and anti-inflammatory factors (IL-4, IL-10, and IFN-γ) were reduced (Fig. 6d).

Overall, these findings suggest that, in the C57BL/6 strain, CD43 deficiency results in an initial reduction in specific pulmonary pro-inflammatory cytokines, followed by a shift toward an anti-inflammatory profile, and a late-stage rebound in inflammation, a cytokine landscape that modifies the immune response, likely contributing to better survival.

### CD43 Deficiency Consistently Reduces Inflammatory Cell Accumulation in the Lungs of C57BL/6 Mice, Similar to BALB/c Mice

**Fig. 7:**
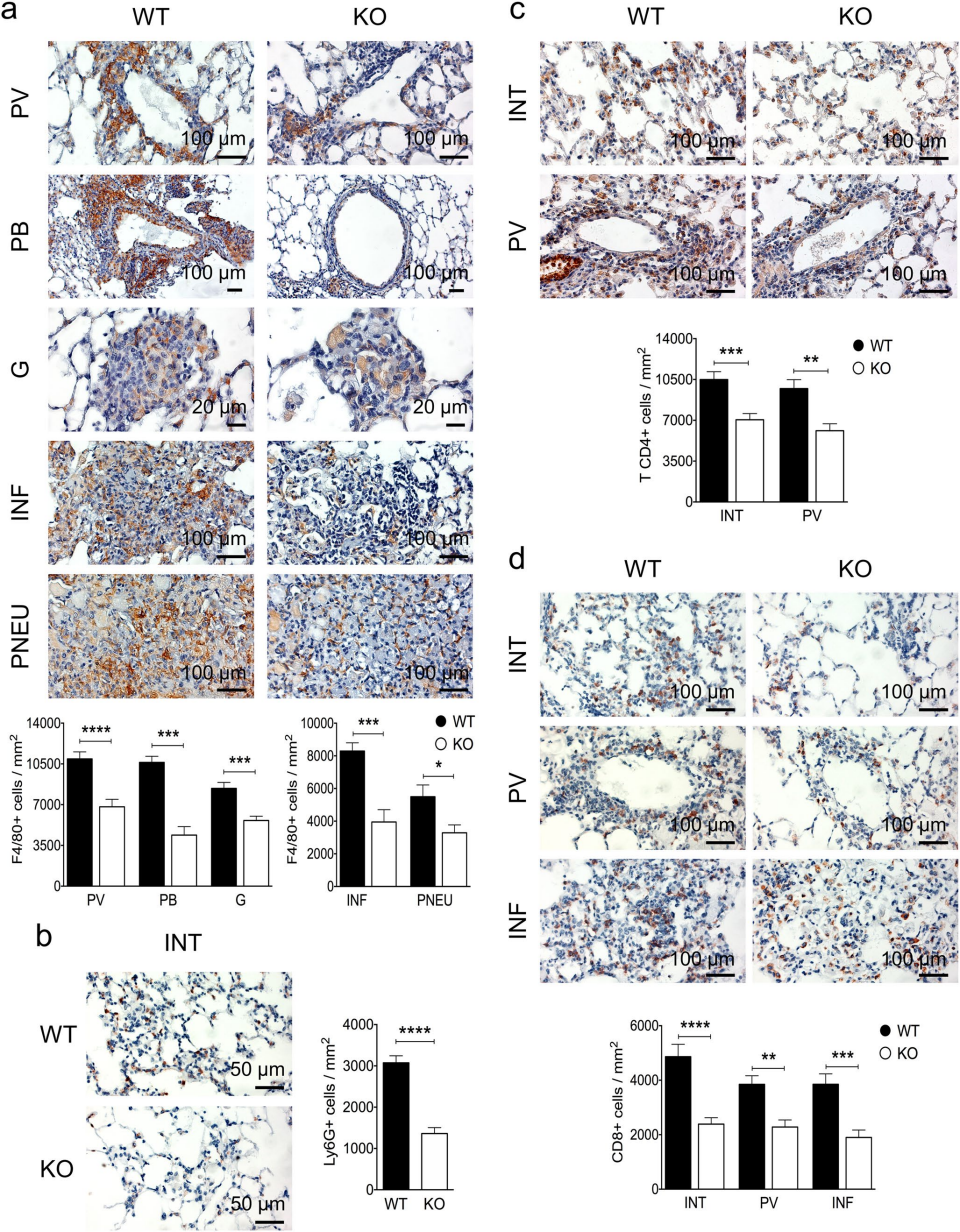
CD43 deficiency reduces pulmonary immune cell infiltration in C57BL/6 mice with late-stage TB in a cell-specific and compartment-specific pattern. Representative immunohistochemistry images of differentially abundant pulmonary immune cell populations (brown staining) in C57BL/6 WT and CD43KO mice at 60 d.p.i., in areas of alveolar-capillary interstitial (INT, 20X), perivascular (PV, 20X), and peribronchial (PB, 10X) inflammatory infiltrate, areas of high cellular infiltration (INF, 20X), pneumonia (PNEU, 20X), and granulomas (G, 63X). Plots show the quantification (cells/mm²) of immune cell populations in manually annotated regions of lung sections. (**a**) Macrophages (F4/80+). (**b**) Neutrophils (Ly6G+). (**c**) CD4 + T cells. (**d**) CD8 + T cells. *n* = 5 mice/group. Statistical analysis: two-tailed Mann-Whitney U test; *p < 0.05, **p < 0.01, ***p < 0.001, ****p < 0.0001. Error bars: SEM

To assess whether the CD43-dependent changes in immune cell populations observed in BALB/c mice also occurred in C57BL/6 mice, we evaluated pulmonary macrophages, neutrophils, and CD4 and CD8 T lymphocytes at 60 d.p.i., the peak of inflammatory infiltration. In line with this strain’s Th1-biased response, macrophages dominated perivascular, peribronchial, highly infiltrated and pneumonic regions, and granulomas in both WT and CD43KO mice. Notably, C57BL/6 CD43KO mice had 1.5 to 2-fold fewer macrophages across all lung compartments, compared to WT mice (Fig. 7a), similar to BALB/c CD43KO mice at 21 d.p.i. Macrophages in the pneumonic regions of C57BL/6 CD43KO mice also showed reduced or absent F4/80 expression (Fig. 7a), as did those of late-stage BALB/c CD43KO mice.

In contrast to BALB/c mice, where neutrophils prevailed in pneumonic areas during late TB, in C57BL/6 mice, they concentrated in alveolar inflammatory infiltrates and densely infiltrated regions, with C57BL/6 CD43KO mice showing a 2-fold reduction in alveolar neutrophils compared to WT mice (Fig. 7b), but not in highly infiltrated pulmonary regions (Fig. S11a).

As in BALB/c mice during late TB, CD4 T cells localized in the alveolar-capillary and perivascular inflammatory infiltrates, densely infiltrated areas throughout the lung, and granulomas (Figs. 7c and S11b). C57BL/6 CD43KO mice had about 30% fewer CD4 T cells in the alveolar-capillary interstitial and perivascular inflammatory infiltrates than WT controls (Fig. 7c). CD8 T cells were mainly found in the perivascular infiltrates, granulomas, inflamed alveolar zones, and densely infiltrated pulmonary regions. CD43 deficiency resulted in an approximate 50% reduction in CD8 T cells in all these compartments, except granulomas, compared with WT mice (Fig. 7d and S11c).

These data indicate that CD43 deficiency modifies the pulmonary immune landscape in C57BL/6 mice in a manner distinct yet functionally similar to that observed in BALB/c mice. In C57BL/6 mice, the absence of CD43 results in a marked decrease in macrophages across all pulmonary compartments, mirroring the early reduction found in BALB/c mice, along with a decrease in interstitial neutrophils and CD4 T cells. While the effect on CD4 T cells is strain-dependent, CD43 is consistently required for the presence of perivascular CD8 T cells in both BALB/c and C57BL/6 mice. As in BALB/c CD43KO mice, the reduced density of these key immune populations within interstitial and perivascular zones aligns with the reduced immunopathology in C57BL/6 CD43KO mice. Taken together, these findings indicate that, despite strain-specific variations in the immune microenvironment, CD43 consistently drives the accumulation of inflammatory cells in lung compartments associated with tissue damage, impairing host survival.

## Discussion

TB remains a global health crisis, underscoring the need to better understand its pathogenesis. The host’s pre-existing immune response, shaped by a complex interplay of genetic and environmental factors, plays a critical role in determining susceptibility to Mtb infection and disease progression [63, 64]. CD43 has been shown to regulate diverse inflammatory processes in multiple disease contexts [25, 26, 29, 30, 32, 65–68]. Its role in TB has been explored primarily in Th1-biased C57BL/6 mice, where in vitro macrophage infection and an in vivo low-dose challenge model demonstrated that, by interacting with Mtb Cpn60.2, CD43 is important for effective macrophage-Mtb binding, immunological responsiveness, and restriction of bacterial burden [22, 25, 26, 28, 30, 41].

Our investigation builds on this knowledge by comparing the role of CD43 in progressive TB infection [50] in two different immunological backgrounds: pro-inflammatory (C57BL/6) and anti-inflammatory (BALB/c) mice. Using WT and CD43KO mice, we found that CD43 regulates the delicate balance between protective immunity and pathological inflammation throughout disease progression, regardless of strain. CD43 deficiency modulates the pulmonary cytokine profile and the abundance of inflammatory cells in key lung compartments, reducing inflammation-related tissue damage and improving survival in both mouse strains while affecting bacterial load control in a strain-dependent manner.

Consistent with CD43 serving as a receptor for Mtb [28, 41], both BALB/c and C57BL/6 CD43KO mice exhibited a lower pulmonary bacterial load during early TB. However, bacterial loads matched those of WT mice by 21 d.p.i. in BALB/c mice and by 120 d.p.i. in C57BL/6 mice, consistent with impaired TNF-α-mediated apoptosis and a faster bacterial doubling time reported for CD43-deficient macrophages infected with bacteria [25, 26].

In both strains, CD43 deficiency enhanced pulmonary pro- and anti-inflammatory cytokines as well as Th17-related cytokine production, at time points when bacterial burden had reached WT levels. In BALB/c CD43KO mice, this was followed by a late-phase reduction in pro-inflammatory cytokines and sustained elevated Th17-related and anti-inflammatory cytokines, correlating with diminished pneumonia. Conversely, in C57BL/6 CD43KO mice, the increase in pulmonary cytokines was preceded by a decline in most pro-inflammatory cytokines, accompanied by increases in IL-17A and anti-inflammatory cytokines, consistent with reduced inflammatory infiltration at 60 d.p.i. This suggests that the survival benefit provided by CD43 deficiency is primarily immunomodulatory rather than dependent on bacterial restriction.

Despite the role of CD43 in cell migration [57–62], its absence did not affect the overall spatial distribution of macrophages, neutrophils, or CD4 and CD8 T cells in the lungs of either strain. However, at the peak of inflammatory infiltration (21 d.p.i., BALB/c; 60 d.p.i., C57BL/6), CD43KO mice showed a significant reduction in macrophages and neutrophils within alveolar interstitial and perivascular regions, which are critical sites for the amplification of inflammation and the development of pneumonia. In C57BL/6 CD43KO mice, the reduction in macrophage numbers extended across all inflammatory regions.

Interestingly, CD43KO mice from both strains shared a common macrophage phenotype in pneumonic regions, characterized by reduced or absent F4/80 expression, suggestive of a predominance of immunosuppressive resident alveolar macrophages over recruited inflammatory monocyte-derived macrophages [69]. Since monocyte-derived macrophages provide a permissive niche for long-term persistence of mycobacteria, as T cells more effectively restrict Mtb replication in resident alveolar macrophages [70], the abundance of monocyte-derived macrophages in the pneumonic areas of BALB/c WT mice may contribute to their elevated bacterial burden during the later stages.

T cell dynamics followed a strain-specific modulation: BALB/c CD43KO mice showed an early increase in interstitial CD4 T cells followed by a late decrease in CD8 T cells within the perivascular, peribronchial, and pneumonic areas, whereas C57BL/6 CD43KO mice showed a coordinated late reduction in both CD4 and CD8 T cells within the interstitial and perivascular zones. Collectively, these findings suggest that by limiting the compartment-specific accumulation of specific inflammatory cells, CD43 deficiency confers protection, and position CD43 as a selective regulator of immune cell migration and homing that influences key anti-Mtb processes, such as recruitment, phagocytosis, and cytokine signaling across different lung microenvironments.

The response of BALB/c and C57BL/6 mouse strains to mycobacterial infections differs in a context-dependent manner. While both mouse strains are highly resistant to Mtb infection [71] and control early Mtb infection in a similar way, BALB/c mice show a reduced capacity to contain the bacteria at late stages of infection, associated with macrophage phenotype [72]. BALB/c mice are also more susceptible to *Mycobacterium avium* infection, linked to increased IL-10 production, and to pulmonary BCG infection, due to a weaker adaptive Th1 response without a compensatory increase in Th2 responses [73, 74]. Notably, despite this predisposition, BALB/c mice mount a stronger pulmonary innate pro-inflammatory response to BCG than C57BL/6 mice [74], while C57BL/6 mice display a greater early innate capacity to control BCG infection when challenged through a different route [75]. Both mouse strains show comparable protection against *Mycobacterium bovis* infection following BCG immunization, with BALB/c mice developing stronger pulmonary Th1 and Th17 responses and C57BL/6 mice exhibiting higher IL-10 production [76]. Although influenced by experimental factors, these evidences underscore that while the host genetics shapes the response, it does not necessarily lead to a fixed outcome, as illustrated by the divergent yet ultimately protective effects of CD43 deficiency across both backgrounds.

While our study offers a comprehensive analysis of the pulmonary environment in CD43-deficient mice, it also raises several questions for future investigation. The use of constitutive CD43KO mice makes it difficult to determine the specific contributions of individual immune cell populations to the phenotypes we observed. Although our approach provided valuable spatial context and revealed significant shifts in immune cell abundance, it did not capture their specific inflammatory phenotypes or activation status. Future work should explore the CD43-dependent mechanisms that regulate cytokine networks and immune cell dynamics, how CD43-driven changes in cell abundance affect compartment-specific functions, and the impact of CD43 deficiency on systemic immunity and bacterial dissemination.

Translating these findings to human disease requires careful consideration of both the complex and varied clinical spectrum of TB and the inherent limitations of murine models. Nonetheless, the correlation between specific SNPs in the CD43 gene and disease susceptibility, severity, and survival in patients with tuberculous meningitis [38] provides a valuable clinical bridge, suggesting a role for CD43 as a modulator of TB outcomes in humans. Future studies should aim to correlate CD43 expression levels with different TB clinical states in human cohorts, while rigorously accounting for genetic, environmental, and immunological confounders. Our murine data provide a conceptual framework for this modulation, proposing that an individual’s pre-existing immune state (analogous to the Th1/Th2 bias in our models) could influence how CD43 activity shapes disease. Understanding CD43-related mechanisms could provide novel host-directed strategies to modulate specific inflammatory circuits in TB or other inflammatory diseases.

## Supplementary Information

Below is the link to the electronic supplementary material.

Supplementary Material 1 (PDF 8.89 MB)

## Data Availability

All relevant research data is included in the main figures and the supplementary material.

## References

[1] World Health Organization. 2024. *Global tuberculosis report 2024*. October.

[2] Pai, Madhukar, Marcel A.. Behr, David Dowdy, Keertan Dheda, Maziar Divangahi, Catharina C.. Boehme, Ann Ginsberg, et al. 2016. Tuberculosis. *Nature Reviews Disease Primers* 2: 16076. 10.1038/nrdp.2016.76. 27784885 10.1038/nrdp.2016.76

[3] World Health Organization. 2025. BCG vaccine. 10.1016/j.vaccine.2018.03.009 29609965

[4] Dartois, Véronique.A., and Eric J.. Rubin. 2022. Anti-tuberculosis treatment strategies and drug development: Challenges and priorities. *Nature Reviews Microbiology* 20: 685–701. 10.1038/s41579-022-00731-y. 35478222 10.1038/s41579-022-00731-y PMC9045034

[5] O’Garra, Anne, Paul S.. Redford, Finlay W.. McNab, Chloe I.. Bloom, Robert J.. Wilkinson, and Matthew P.R.. Berry. 2013. The immune response in tuberculosis. *Annual Review of Immunology* 31: 475–527. 10.1146/annurev-immunol-032712-095939. 23516984 10.1146/annurev-immunol-032712-095939

[6] Ravesloot-Chávez, Mariëtta M.., Erik Van Dis, and Sarah A.. Stanley. 2021. The innate immune response to *Mycobacterium tuberculosis* infection. *Annual Review of Immunology* 39: 611–637. 10.1146/annurev-immunol-093019-010426. 33637017 10.1146/annurev-immunol-093019-010426

[7] Vats, Deepak, Geeta Rani, Alisha Arora, Vidushi Sharma, Isha Rathore, Shaikh Abdul Mubeen, and Archana Singh. 2024. Tuberculosis and T cells: Impact of T cell diversity in tuberculosis infection. *Tuberculosis (Edinburgh, Scotland)* 149: 102567. 10.1016/j.tube.2024.102567. 39305817 10.1016/j.tube.2024.102567

[8] Sasindran, Smitha J.., and Jordi B.. Torrelles. 2011. *Mycobacterium tuberculosis* infection and inflammation: What is beneficial for the host and for the bacterium? *Frontiers in Microbiology*. 10.3389/fmicb.2011.00002. 21687401 10.3389/fmicb.2011.00002 PMC3109289

[9] Guirado, Evelyn, Larry S.. Schlesinger, and Gilla Kaplan. 2013. Macrophages in tuberculosis: Friend or foe. *Seminars in Immunopathology* 35: 563–583. 10.1007/s00281-013-0388-2. 23864058 10.1007/s00281-013-0388-2 PMC3763202

[10] Queval, Christophe J.., Roland Brosch, and Roxane Simeone. 2017. The macrophage: A disputed fortress in the battle against *Mycobacterium tuberculosis*. *Frontiers in Microbiology*. 10.3389/fmicb.2017.02284. 29218036 10.3389/fmicb.2017.02284 PMC5703847

[11] Yang, Chao-Tsung., C.J. Cambier, J. Muse. Davis, Christopher J.. Hall, Philip S.. Crosier, and Lalita Ramakrishnan. 2012. Neutrophils exert protection in the early tuberculous granuloma by oxidative killing of mycobacteria phagocytosed from infected macrophages. *Cell Host & Microbe* 12: 301–312. 10.1016/j.chom.2012.07.009. 22980327 10.1016/j.chom.2012.07.009 PMC3638950

[12] Kroon, Elouise E.., Anna K.. Coussens, Craig Kinnear, Marianna Orlova, Marlo Möller, Allison Seeger, Robert J.. Wilkinson, Eileen G.. Hoal, and Erwin Schurr. 2018. Neutrophils: Innate effectors of TB resistance? *Frontiers in Immunology*. 10.3389/fimmu.2018.02637. 30487797 10.3389/fimmu.2018.02637 PMC6246713

[13] Muefong, Caleb N.., and Jayne S.. Sutherland. 2020. Neutrophils in tuberculosis-associated inflammation and lung pathology. *Frontiers in Immunology* 11: 962. 10.3389/fimmu.2020.00962. 32536917 10.3389/fimmu.2020.00962 PMC7266980

[14] Alcantara, Cheldon Ann, Ira Glassman, Kevin H.. Nguyen, Arpitha Parthasarathy, and Vishwanath Venketaraman. 2023. Neutrophils in *Mycobacterium tuberculosis*. *Vaccines*. 10.3390/vaccines11030631. 36992214 10.3390/vaccines11030631 PMC10055476

[15] Jasenosky, Luke D.., Thomas J.. Scriba, Willem A.. Hanekom, and Anne E.. Goldfeld. 2015. T cells and adaptive immunity to *Mycobacterium tuberculosis* in humans. *Immunological Reviews* 264: 74–87. 10.1111/imr.12274. 25703553 10.1111/imr.12274

[16] Lin, Philana Ling, and Jo Anne L.. Flynn. 2015. CD8 T cells and *Mycobacterium tuberculosis* infection. *Seminars in Immunopathology* 37: 239–249. 10.1007/s00281-015-0490-8. 25917388 10.1007/s00281-015-0490-8 PMC4439333

[17] Morgan, Jeffrey, Kaylin Muskat, Rashmi Tippalagama, Alessandro Sette, Julie Burel, and Cecilia S.. Lindestam Arlehamn. 2021. Classical CD4 T cells as the cornerstone of antimycobacterial immunity. *Immunological Reviews* 301: 10–29. 10.1111/imr.12963. 33751597 10.1111/imr.12963 PMC8252593

[18] Cicchese, Joseph M.., Stephanie Evans, Caitlin Hult, Louis R.. Joslyn, Timothy Wessler, Jess A.. Millar, Simeone Marino, et al. 2018. Dynamic balance of pro- and anti-inflammatory signals controls disease and limits pathology. *Immunological Reviews* 285: 147–167. 10.1111/imr.12671. 30129209 10.1111/imr.12671 PMC6292442

[19] Liu, Cui Hua, Haiying Liu, and Baoxue Ge. 2017. Innate immunity in tuberculosis: Host defense vs pathogen evasion. *Cellular and Molecular Immunology* 14: 963–975. 10.1038/cmi.2017.88. 10.1038/cmi.2017.88 PMC5719146 28890547

[20] Montero, Paula, Inés. Roger, Javier Milara, and Julio Cortijo. 2025. Immunomodulatory properties of transmembrane mucins: From chronic diseases to cancer. *Physiological Reviews* 105: 2233–2304. 10.1152/physrev.00034.2024. 40465524 10.1152/physrev.00034.2024

[21] Hartshorn, K.L., L.S. Liou, M.R. White, M.M. Kazhdan, J.L. Tauber, and A.I. Tauber. 1995. Neutrophil deactivation by influenza A virus. Role of hemagglutinin binding to specific sialic acid-bearing cellular proteins. *The Journal of Immunology* 154: 3952–3960. 7706733

[22] Fratazzi, Candida, Robert D By, Claudio Arbeit, Thomas A Carini, Blair Gerken, Eileen Remold-O. Ardman, and G Remold Heinz. 2000. A Macrophage Invasion Mechanism for Mycobacteria Implicating the Extracellular Domain of CD43. The Journal of Experimental Medicine 192. 10.1084/jem.192.2.183 PMC2193255 10899905

[23] Ruhl, Stefan, John O.. Cisar, and Ann L.. Sandberg. 2000. Identification of Polymorphonuclear Leukocyte and HL-60 Cell Receptors for Adhesins of *Streptococcus gordonii* and *Actinomyces naeslundii*. *Infection and Immunity* 68: 6346–6354. 10.1128/IAI.68.11.6346-6354.2000. 10.1128/iai.68.11.6346-6354.2000 PMC97718 11035744

[24] Todeschini, Adriane R.., Murielle F.. Girard, Jean-Michel. Wieruszeski, Marise P.. Nunes, George A.. DosReis, Lúcia. Mendonça-Previato, and José O.. Previato. 2002. *trans*-Sialidase from *Trypanosoma cruzi*Binds Host T-lymphocytes in a Lectin Manner*. *Journal of Biological Chemistry* 277: 45962–45968. 10.1074/jbc.M203185200. 10.1074/jbc.M203185200 12237289

[25] Randhawa, April K., Hermann J. Ziltener, Jasmeen S. Merzaban, and Richard W. Stokes. 2005. *CD43 Is Required for Optimal Growth Inhibition of Mycobacterium tuberculosis in Macrophages and in Mice 1*. *The Journal of Immunology*. 10.4049/jimmunol.175.3.1805. 10.4049/jimmunol.175.3.1805 16034122

[26] Randhawa, April K.., Hermann J.. Ziltener, and Richard W.. Stokes. 2008. CD43 controls the intracellular growth of Mycobacterium tuberculosis through the induction of TNF-α-mediated apoptosis. *Cellular Microbiology* 10: 2105–2117. 10.1111/j.1462-5822.2008.01194.x. 18637079 10.1111/j.1462-5822.2008.01194.x

[27] Urano-Tashiro, Yumiko, Ayako Yajima, Eizo Takashima, Yukihiro Takahashi, and Kiyoshi Konishi. 2008. Binding of the *Streptococcus gordonii* DL1 surface protein Hsa to the host cell membrane glycoproteins CD11b, CD43, and CD50. *Infection and Immunity* 76: 4686–4691. 10.1128/IAI.00238-08. 18678668 10.1128/IAI.00238-08 PMC2546842

[28] Hickey, Tyler B.M.., Hermann J.. Ziltener, David P.. Speert, and Richard W.. Stokes. 2010. Mycobacterium tuberculosis employs Cpn60.2 as an adhesin that binds CD43 on the macrophage surface. *Cellular Microbiology* 12: 1634–1647. 10.1111/j.1462-5822.2010.01496.x. 20633027 10.1111/j.1462-5822.2010.01496.x

[29] Nico, Dirlei, Naiara Maran, Leonardo Santos, Erivan Schnaider Ramos-Junior, Natália Rodrigues. Mantuano, Joseane Lima Prado. Coutinho, Andre Macedo Vale, et al. 2015. Expression of leukosialin (CD43) defines a major intrahepatic T cell subset associated with protective responses in visceral leishmaniasis. *Parasites and Vectors*. 10.1186/s13071-015-0721-9. 10.1186/s13071-015-0721-9 PMC4340829 25874567

[30] Torres-Huerta, Alvaro, Tomás Villaseñor, Angel Flores-Alcantar, Cristina Parada, Estefanía Alemán-Navarro, Clara Espitia, Gustavo Pedraza-Alva, and Yvonne Rosenstein. 2017. Interaction of the CD43 Sialomucin with the Mycobacterium tuberculosis Cpn60.2 Chaperonin Leads to Tumor Necrosis Factor Alpha Production. *Infection and Immunity* 85: e00915-16. 10.1128/IAI.00915-16. 10.1128/IAI.00915-16 PMC5328480 28069816

[31] Jans, Jop, Wendy W.J.. Unger, Marloes Vissers, Inge M.L.. Ahout, Inge Schreurs, Arthur Wickenhagen, Ronald De Groot, Marien I.. De Jonge, and Gerben Ferwerda. 2018. Siglec-1 inhibits RSV‐induced interferon gamma production by adult T cells in contrast to newborn T cells. *European Journal of Immunology* 48: 621–631. 10.1002/eji.201747161. 29266251 10.1002/eji.201747161 PMC5947594

[32] Alisson-Silva, Frederico, Natália Rodrigues. Mantuano, Ana Luiza Lopes, Andréia Vasconcelos-dos-Santos, André Macedo. Vale, Miriam Maria Costa, Judy L.. Cannon, Ana Carolina Oliveira, and Adriane R.. Todeschini. 2019. CD43 sialoglycoprotein modulates cardiac inflammation and murine susceptibility to Trypanosoma cruzi infection. *Scientific Reports*. 10.1038/s41598-019-45138-7. 10.1038/s41598-019-45138-7 PMC6565700 31197200

[33] Hao, Qin, Suman Kundu, Sreerama Shetty, and Hua Tang. 2024. Runx3 regulates CD8 + T Cell local expansion and CD43 glycosylation in mice by H1N1 Influenza A virus infection. *International Journal of Molecular Sciences* 25: 4220. 10.3390/ijms25084220. 38673806 10.3390/ijms25084220 PMC11050410

[34] Kawano, Sae, Chisaki Noda, Saotomo Itoh, Ayaka Urabe, Chifumi Fujii, Isamu Ogawa, Ryo Suzuki, and Shigeaki Hida. 2024. Staphylococcal superantigen-like protein 3 triggers murine mast cell adhesion by binding to <sc>CD43</sc> and augments mast cell activation. *Genes to Cells* 29: 397–416. 10.1111/gtc.13111. 38454012 10.1111/gtc.13111

[35] Fierro, Nora A.., Gustavo Pedraza-Alva, and Yvonne Rosenstein. 2006. TCR-dependent cell response is modulated by the timing of CD43 engagement. *The Journal of Immunology* 176: 7346–7353. 10.4049/jimmunol.176.12.7346. 16751378 10.4049/jimmunol.176.12.7346

[36] Torres-Huerta, Alvaro, Estefania Aleman-Navarro, Maria Elena Bravo-Adame, Monserrat Alba Sandoval-Hernandez, Oscar Arturo Migueles-Lozano, and Yvonne Rosenstein. 2018. CD43. In *Encyclopedia of Signaling Molecules*, ed. Sangdun Choi, 893–905. Cham: Springer International Publishing. 10.1007/978-3-319-67199-4_523.

[37] Sandoval-Hernández, Monserrat Alba, Nora Alma Fierro, José Ignacio. Veytia-Bucheli, Den Alejandro Alvarado-Velázquez, Estefanía Alemán-Navarro, Erika Melchy-Pérez, Constance Auvynet, et al. 2024. Differential impact of CD43 and CD28 on T-Cell differentiation depending on the order of engagement with the TCR. *International Journal of Molecular Sciences* 25: 3135. 10.3390/ijms25063135. 10.3390/ijms25063135 PMC10970108 38542109

[38] Campo, Monica, April K.. Randhawa, Sarah Dunstan, Jeremy Farrar, Maxine Caws, Nguyen Duc Bang, Nguyen Ngoc Lan, et al. 2015. Common polymorphisms in the CD43 gene region are associated with tuberculosis disease and mortality. *American Journal of Respiratory Cell and Molecular Biology* 52: 342–348. 10.1165/rcmb.2014-0114OC. 25078322 10.1165/rcmb.2014-0114OC PMC4370260

[39] Sassetti, Christopher M.., Dana H.. Boyd, and Eric J.. Rubin. 2003. Genes required for mycobacterial growth defined by high density mutagenesis. *Molecular Microbiology* 48: 77–84. 10.1046/j.1365-2958.2003.03425.x. 12657046 10.1046/j.1365-2958.2003.03425.x

[40] Hu, Yanmin, Brian Henderson, Peter A.. Lund, Peter Tormay, M. Tabish. Ahmed, Sudagar S.. Gurcha, Gurdyal S.. Besra, and Anthony R.M.. Coates. 2008. A *Mycobacterium tuberculosis* mutant lacking the groEL homologue cpn60.1 is viable but fails to induce an inflammatory response in animal models of infection. *Infection and Immunity* 76: 1535–1546. 10.1128/IAI.01078-07. 18227175 10.1128/IAI.01078-07 PMC2292875

[41] Hickey, Tyler B.M.., Lisa M.. Thorson, David P.. Speert, Mamadou Daffé, and Richard W.. Stokes. 2009. *Mycobacterium tuberculosis* Cpn60.2 and DnaK are located on the bacterial surface, where Cpn60.2 facilitates efficient bacterial association with macrophages. *Infection and Immunity* 77: 3389–3401. 10.1128/IAI.00143-09. 19470749 10.1128/IAI.00143-09 PMC2715658

[42] Barber-Mayer, Katrin D.., and Daniel L.. Barber. 2015. Innate and adaptive cellular immune responses to *Mycobacterium tuberculosis* infection. *Cold Spring Harbor Perspectives in Medicine*. 10.1101/cshperspect.a018424. 10.1101/cshperspect.a018424 PMC4665043 26187873

[43] Sia, Jonathan Kevin, and Jyothi Rengarajan. 2019. Immunology of *Mycobacterium tuberculosis* infections. *Microbiology Spectrum* 7: 7.4.6. 10.1128/microbiolspec.GPP3-0022-2018. 10.1128/microbiolspec.gpp3-0022-2018 PMC6636855 31298204

[44] Rook, Graham A.W.., Rogelio Hernández-Pando, and Alimuddin Zumla. 2009. Tuberculosis due to high-dose challenge in partially immune individuals: A problem for vaccination? *Journal of Infectious Diseases* 199: 613–618. 10.1086/596654. 19199549 10.1086/596654

[45] Witebsky, Frank G., and Patricia Kruczak-Filipov. 1996. Identification of mycobacteria by conventional methods. *Clinical Micobacteriology* 16. 8866181

[46] Forbes, Betty A, S. Geraldine, Melissa B Hall, Susan M Miller, Marie-Claire Novak, Max Rowlinson, David M Salfinger, Akos Somoskövi, and Warshauer. 2018. and Michael L Wilson. Practice Guidelines for Clinical Microbiology Laboratories: Mycobacteria. 10.1128/CMR 10.1128/CMR.00038-17 PMC5967691 29386234

[47] Soldevilla, Pablo, Cristina Vilaplana, and Cardona Pere Joan. 2023. Mouse Models for Mycobacterium tuberculosis Pathogenesis: Show and Do Not Tell. *Pathogens* 12. MDPI. 10.3390/pathogens12010049 10.3390/pathogens12010049 PMC9865329 36678397

[48] Gupta, Manish, Geetha Srikrishna, Sabra L.. Klein, and William R.. Bishai. 2022. Genetic and hormonal mechanisms underlying sex-specific immune responses in tuberculosis. *Trends in Immunology* 43: 640–656. 10.1016/j.it.2022.06.004. 35842266 10.1016/j.it.2022.06.004 PMC9344469

[49] Park, John K, Yvonne J Rosenstein, Eileen Remold-O’Donnell, and Fred S Rosen. 1991. Enhancement of T-cell activation by the CD43 molecule whose expression is defective in Wiskott-Aidrich syndrome 350. 10.1038/350706a0 10.1038/350706a0 2023632

[50] Hernandez-Pando, R., H. Orozcoe, A. Sampieri, L. Pavon, C. Velasquillo, J. Larriva-Sahd, J. M. Alcocer$, and M. V. Madrid. 1996. Correlation between the kinetics of Thl/Th2 cells and pathology in a murine model of experimental pulmonary tuberculosis. Immunology. Vol. 89. PMC1456655 8911136

[51] Castañon-Arreola, Mauricio, Yolanda López-Vidal, Clara Espitia-Pinzón, and Rogelio Hernández-Pando. 2005. A new vaccine against tuberculosis shows greater protection in a mouse model with progressive pulmonary tuberculosis. *Tuberculosis* 85: 115–126. 10.1016/j.tube.2004.10.004. 15687035 10.1016/j.tube.2004.10.004

[52] Bankhead, Peter, Maurice B.. Loughrey, José A.. Fernández, Yvonne Dombrowski, Darragh G.. McArt, Philip D.. Dunne, Stephen McQuaid, et al. 2017. QuPath: Open source software for digital pathology image analysis. *Scientific Reports* 7: 16878. 10.1038/s41598-017-17204-5. 29203879 10.1038/s41598-017-17204-5 PMC5715110

[53] Lyadova, I.V., and A.V. Panteleev. 2015. Th1 and Th17 cells in tuberculosis: Protection, pathology, and biomarkers. *Mediators of Inflammation*. 10.1155/2015/854507. 26640327 10.1155/2015/854507 PMC4657112

[54] Domingo-Gonzalez, Racquel, Oliver Prince, Andrea Cooper, and Shabaana A.. Khader. 2016. Cytokines and chemokines in *Mycobacterium tuberculosis* infection. *Microbiology Spectrum*. 10.1128/microbiolspec.tbtb2-0018-2016. 27763255 10.1128/microbiolspec.TBTB2-0018-2016 PMC5205539

[55] Orme, Ian M., Richard T. Robinson, and Andrea M. Cooper. 2015. The balance between protective and pathogenic immune responses in the TB-infected lung. *Nature Immunology* 16: 57–63. 10.1038/ni.3048. 25521685 10.1038/ni.3048

[56] Chandra, Pallavi, Steven J.. Grigsby, and Jennifer A.. Philips. 2022. Immune evasion and provocation by *Mycobacterium tuberculosis*. *Nature Reviews Microbiology* 20: 750–766. 10.1038/s41579-022-00763-4. 35879556 10.1038/s41579-022-00763-4 PMC9310001

[57] Rosenstein, Yvonne, Angelica Santana, and Gustavo Pedraza-Alva. 1999. CD43, a molecule with multiple functions. *Immunologic Research* 20: 89–99. 10.1007/BF02786465. 10580634 10.1007/BF02786465

[58] Van Den Berg, Timo K., Deepa Nath, Hermann J. Ziltener, Dietmar Vestweber, Minoru Fukuda, Irma Van Die, and Paul R. Crocker. 2001. Cutting edge: CD43 functions as a T cell counterreceptor for the macrophage adhesion receptor Sialoadhesin (Siglec-1). *The Journal of Immunology* 166: 3637–3640. 10.4049/jimmunol.166.6.3637. 11238599 10.4049/jimmunol.166.6.3637

[59] Matsumoto, Masanori, Kazuyuki Atarashi, Eiji Umemoto, Yuko Furukawa, Akiko Shigeta, Masayuki Miyasaka, and Takako Hirata. 2005. CD43 functions as a ligand for E-Selectin on activated T cells. *The Journal of Immunology* 175: 8042–8050. 10.4049/jimmunol.175.12.8042. 16339541 10.4049/jimmunol.175.12.8042

[60] Matsumoto, Masanori, Akiko Shigeta, Masayuki Miyasaka, and Takako Hirata. 2008. CD43 plays both antiadhesive and proadhesive roles in neutrophil rolling in a context-dependent manner. *The Journal of Immunology* 181: 3628–3635. 10.4049/jimmunol.181.5.3628. 18714037 10.4049/jimmunol.181.5.3628

[61] Moreno-Amaral, Andrea N., Evelyne Gout, Claudia Danella-Polli, Fanny Tabarin, Philippe Lesavre, Gabriela Pereira-da-Silva, Nicole M. Thielens, and Lise Halbwachs-Mecarelli. 2011. M-ficolin and leukosialin (CD43): New partners in neutrophil adhesion. *Journal of Leukocyte Biology* 91: 469–474. 10.1189/jlb.0911460. 22167719 10.1189/jlb.0911460

[62] Velázquez, Francisco, Anna Grodecki-Pena, Andrew Knapp, Ane M. Salvador, Tania Nevers, Kevin J. Croce, and Pilar Alcaide. 2016. CD43 functions as an E-Selectin ligand for Th17 cells in vitro and is required for rolling on the vascular endothelium and Th17 cell recruitment during inflammation in vivo. *The Journal of Immunology* 196: 1305–1316. 10.4049/jimmunol.1501171. 26700769 10.4049/jimmunol.1501171 PMC4724552

[63] Kumari, Priyanka, and Laxman S.. Meena. 2014. Factors affecting susceptibility to *Mycobacterium tuberculosis*: A close view of immunological defence mechanism. *Applied Biochemistry and Biotechnology* 174: 2663–2673. 10.1007/s12010-014-1217-3. 25296626 10.1007/s12010-014-1217-3

[64] Simmons, Jason D.., Catherine M.. Stein, Chetan Seshadri, Monica Campo, Galit Alter, Sarah Fortune, Erwin Schurr, et al. 2018. Immunological mechanisms of human resistance to persistent *Mycobacterium tuberculosis* infection. *Nature Reviews Immunology* 18: 575–589. 10.1038/s41577-018-0025-3. 29895826 10.1038/s41577-018-0025-3 PMC6278832

[65] Onami, Thandi M.., Laurie E.. Harrington, Matthew A.. Williams, Marisa Galvan, Christian P.. Larsen, Thomas C.. Pearson, N. Manjunath, Linda G.. Baum, Brad D.. Pearce, and Rafi Ahmed. 2002. Dynamic regulation of T cell immunity by CD43. *The Journal of Immunology* 168: 6022–6031. 10.4049/jimmunol.168.12.6022. 12055210 10.4049/jimmunol.168.12.6022

[66] Ford, Mandy L.., Thandi M.. Onami, Anne I.. Sperling, Rafi Ahmed, and Brian D.. Evavold. 2003. CD43 modulates severity and onset of experimental autoimmune encephalomyelitis. *The Journal of Immunology* 171: 6527–6533. 10.4049/jimmunol.171.12.6527. 14662853 10.4049/jimmunol.171.12.6527

[67] Ford, Mandy L.., and Brian D.. Evavold. 2006. Modulation of MOG 37-50-specific CD8 + T cell activation and expansion by CD43. *Cellular Immunology* 240: 53–61. 10.1016/j.cellimm.2006.06.007. 16890924 10.1016/j.cellimm.2006.06.007

[68] Fay, Katherine T.., Deena B.. Chihade, Ching Wen Chen, Nathan J.. Klingensmith, John D.. Lyons, Kimberly Ramonell, Zhe Liang, Craig M.. Coopersmith, and Mandy L.. Ford. 2018. Increased mortality in CD43-deficient mice during sepsis. *PLoS ONE*. 10.1371/journal.pone.0202656. 30226896 10.1371/journal.pone.0202656 PMC6143188

[69] Kloc, Malgorzata. 2017. Macrophages: origin, functions and biointervention. Results and Problems in Cell Differentiation volume 62. Cham: Springer.

[70] Lai, Rocky, Travis Williams, Tasfia Rakib, Jinhee Lee, and Samuel M.. Behar. 2024. Heterogeneity in lung macrophage control of *Mycobacterium tuberculosis* is modulated by T cells. *Nature Communications* 15: 5710. 10.1038/s41467-024-48515-7. 38977711 10.1038/s41467-024-48515-7 PMC11231272

[71] Medina, North. 1998. Resistance ranking of some common inbred mouse strains to Mycobacterium tuberculosis and relationship to major histocompatibility complex haplotype and Nramp1 genotype. *Immunology* 93:270–274. 10.1046/j.1365-2567.1998.00419.x 9616378 10.1046/j.1365-2567.1998.00419.x PMC1364188

[72] Bertolini, Thais Barboza, Alexandre Ignacio De Souza, Ana Flávia Gembre, Annie Rocio Piñeros, Rafael De Queiroz Prado, João Santana. Silva, Leandra Naira Zambelli. Ramalho, and Vânia Luiza Deperon. Bonato. 2016. Genetic background affects the expansion of macrophage subsets in the lungs of *Mycobacterium tuberculosis* -infected hosts. *Immunology* 148: 102–113. 10.1111/imm.12591. 10.1111/imm.12591 PMC4819146 26840507

[73] Roque, Susana, Claudia Nobrega, Rui Appelberg, and Margarida Correia-Neves. 2007. IL-10 underlies distinct susceptibility of BALB/c and C57BL/6 mice to *Mycobacterium avium* infection and influences efficacy of antibiotic therapy. *The Journal of Immunology* 178: 8028–8035. 10.4049/jimmunol.178.12.8028. 10.4049/jimmunol.178.12.8028 17548640

[74] Wakeham, Julia, Jun Wang, and Zhou Xing. 2000. Genetically determined disparate innate and adaptive cell-mediated immune responses to pulmonary *Mycobacterium bovis* BCG infection in C57BL/6 and BALB/c mice. *Infection and Immunity* 68: 6946–6953. 10.1128/IAI.68.12.6946-6953.2000. 11083818 10.1128/iai.68.12.6946-6953.2000 PMC97803

[75] Arko-Mensah, John, Muhammad J.. Rahman, Irene R.. Dégano, Olga D.. Chuquimia, Agathe L.. Fotio, Irene Garcia, and Carmen Fernández. 2009. Resistance to mycobacterial infection: A pattern of early immune responses leads to a better control of pulmonary infection in C57BL/6 compared with BALB/c mice. *Vaccine* 27: 7418–7427. 10.1016/j.vaccine.2009.06.110. 19735756 10.1016/j.vaccine.2009.06.110

[76] Garcia-Pelayo, M. Carmen., Véronique.S. Bachy, Daryan A.. Kaveh, and Philip J.. Hogarth. 2015. BALB/c mice display more enhanced BCG vaccine induced Th1 and Th17 response than C57BL/6 mice but have equivalent protection. *Tuberculosis* 95: 48–53. 10.1016/j.tube.2014.10.012. 25467292 10.1016/j.tube.2014.10.012

