# Supplementary material for: The Sialomucin CD43 Drives Tuberculosis Immunopathology Across Different Host Genetic Backgrounds
Source: Inflammation. 2026 Jun 10;49(1):175. doi: 10.1007/s10753-026-02520-8 (PMC13476292; doi:10.1007/s10753-026-02520-8)
Supplement: Supplementary file 1 [file 10753_2026_2520_MOESM1_ESM.pdf]

Inflammation (Supplementary material)

The sialomucin CD43 drives tuberculosis immunopathology across different host genetic backgrounds

Estefanía Alemán-Navarro[1,2], Dulce A. Mata-Espinosa[3], Manuel O. López-Torres[3], Jorge A. Barrios Payán[3], Ángel F. Flores-Alcantar[1], Erika I. Melchy-Pérez[1], Rogelio Hernández-Pando[3], Yvonne Rosenstein[1*]

[1] Instituto de Biotecnología, Universidad Nacional Autónoma de México (UNAM), Campus Morelos, Cuernavaca, México

[2] Posgrado en Ciencias Bioquímicas, Universidad Nacional Autónoma de México (UNAM), Ciudad de México, México

[3] Instituto Nacional de Ciencias Médicas y Nutrición Salvador Zubirán, Ciudad de México, México.

* Correspondence: yvonne.rosenstein@ibt.unam.mx

Fig. S1

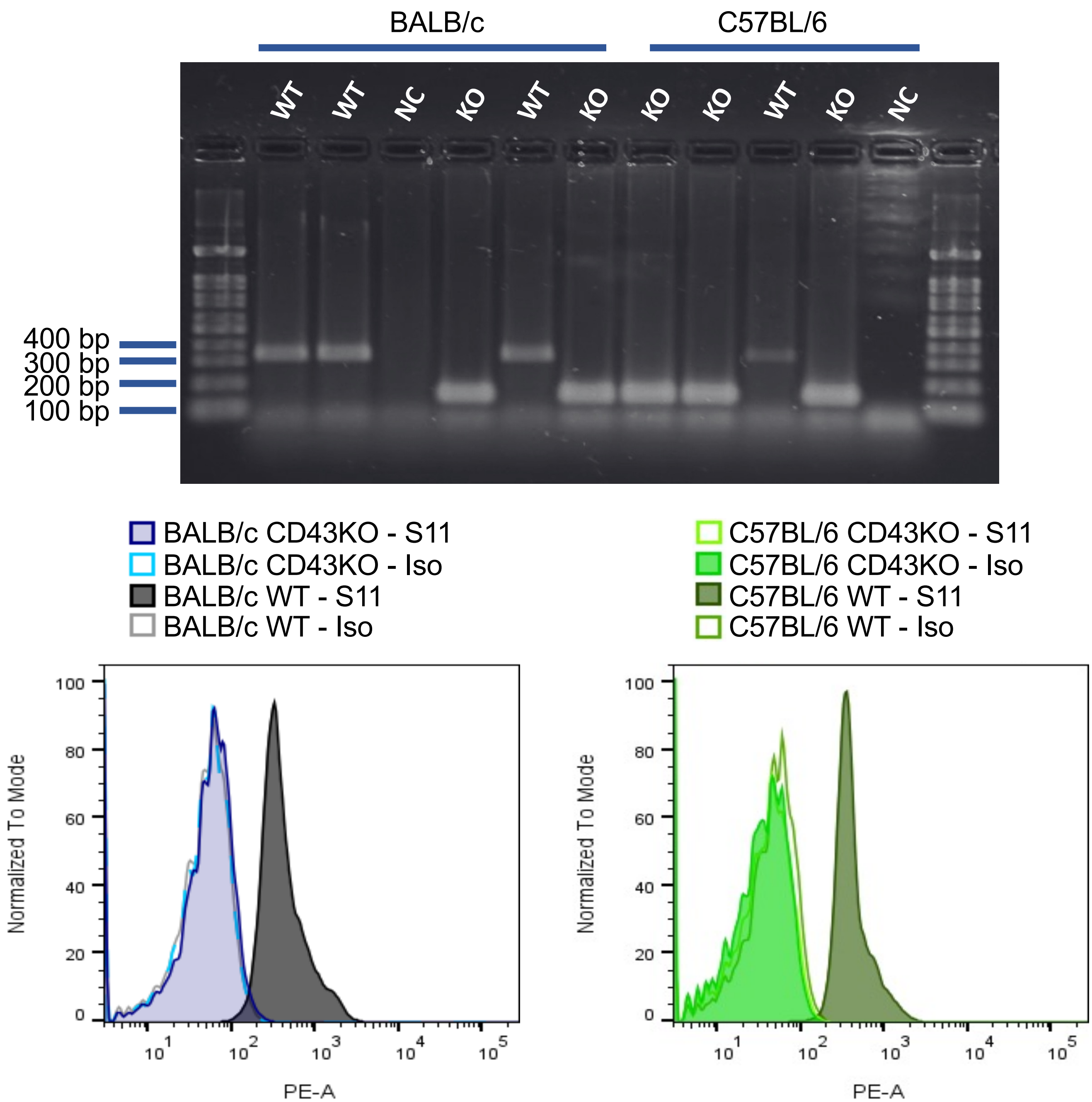

Fig. S1 Genotypification and phenotypification of BALB/c and C57BL/6 CD43KO mice. Upper panel: genotypification of BALB/c and C57BL/6 WT (+/+) and CD43KO (-/-) mice by PCR. For WT mice, the expected band size is 338 bp; for CD43KO mice, in which the CD43 gene is interrupted by a neomycin resistance (Neo) cassette, the expected band size is 200 bp; NC: negative control. Lower panel: representative histograms displaying CD43 expression in thymus cells from WT and CD43KO BALB/c and C57BL/6 mice, stained with anti-murine CD43-PE mAb (clone S11) or an anti-rat IgG2a κ-isotype control (ISO).

Fig. S2

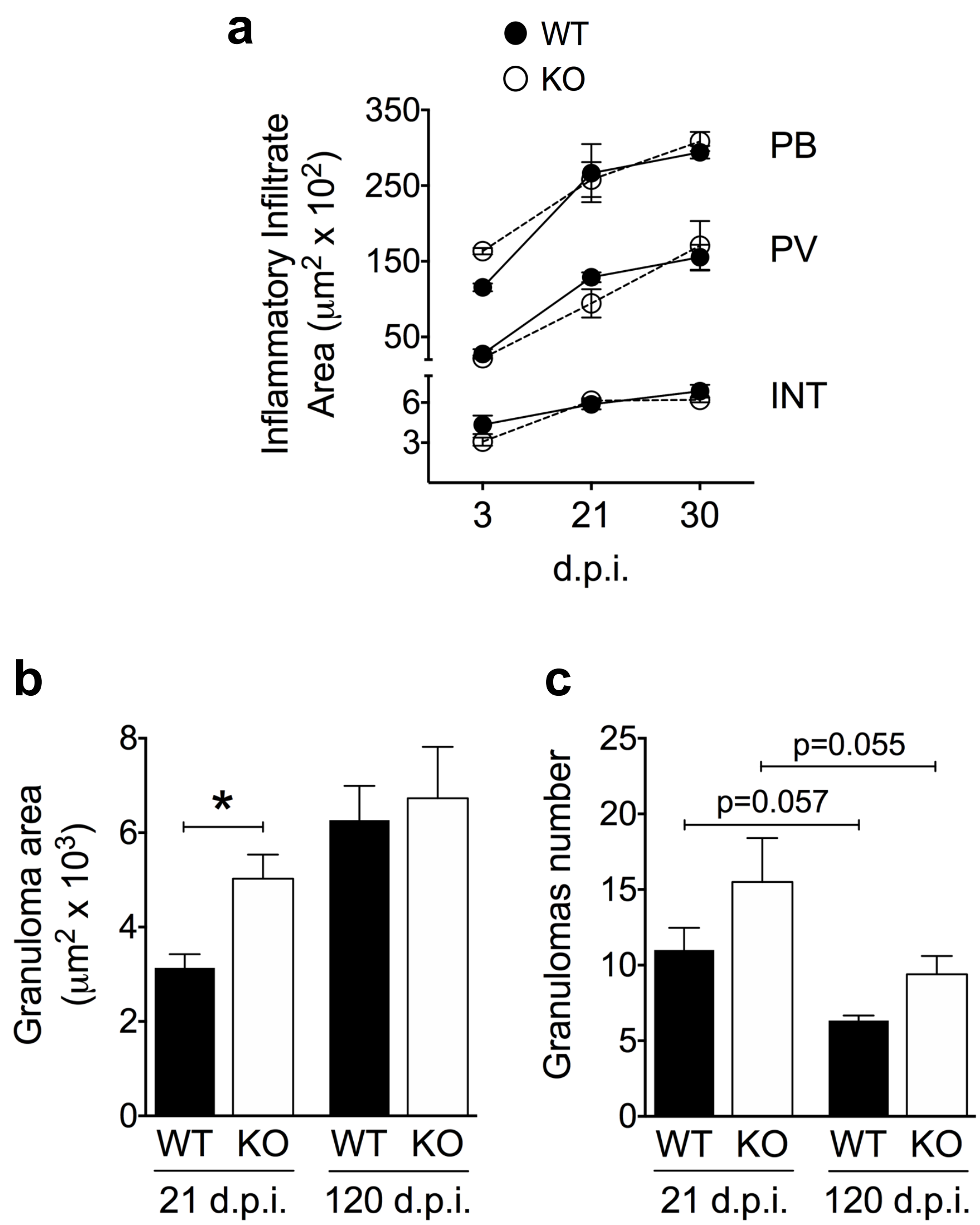

Fig. S2 In BALB/c mice, CD43 deficiency increases granuloma size without affecting inflammatory infiltrate in early TB **a.** Area of alveolar-capillary interstitial (INT), perivascular (PV), and peribronchial (PB) cellular inflammatory infiltrate during early TB (3, 21, and 30 d.p.i.), assessed by morphometric analysis in the lungs of BALB/c WT and CD43KO mice. **b.** Area of single granulomas in the lungs at 21 and 120 d.p.i.; morphometric analysis. **c.** Number of pulmonary granulomas at 21 and 120 d.p.i.; morphometric analysis. n=5 mice/group (days 3 and 30) and n=10 mice/group (days 21 and 120). Statistical analysis: two-tailed Mann-Whitney U test; *p<0.05. Error bars: SEM.

Fig. S3

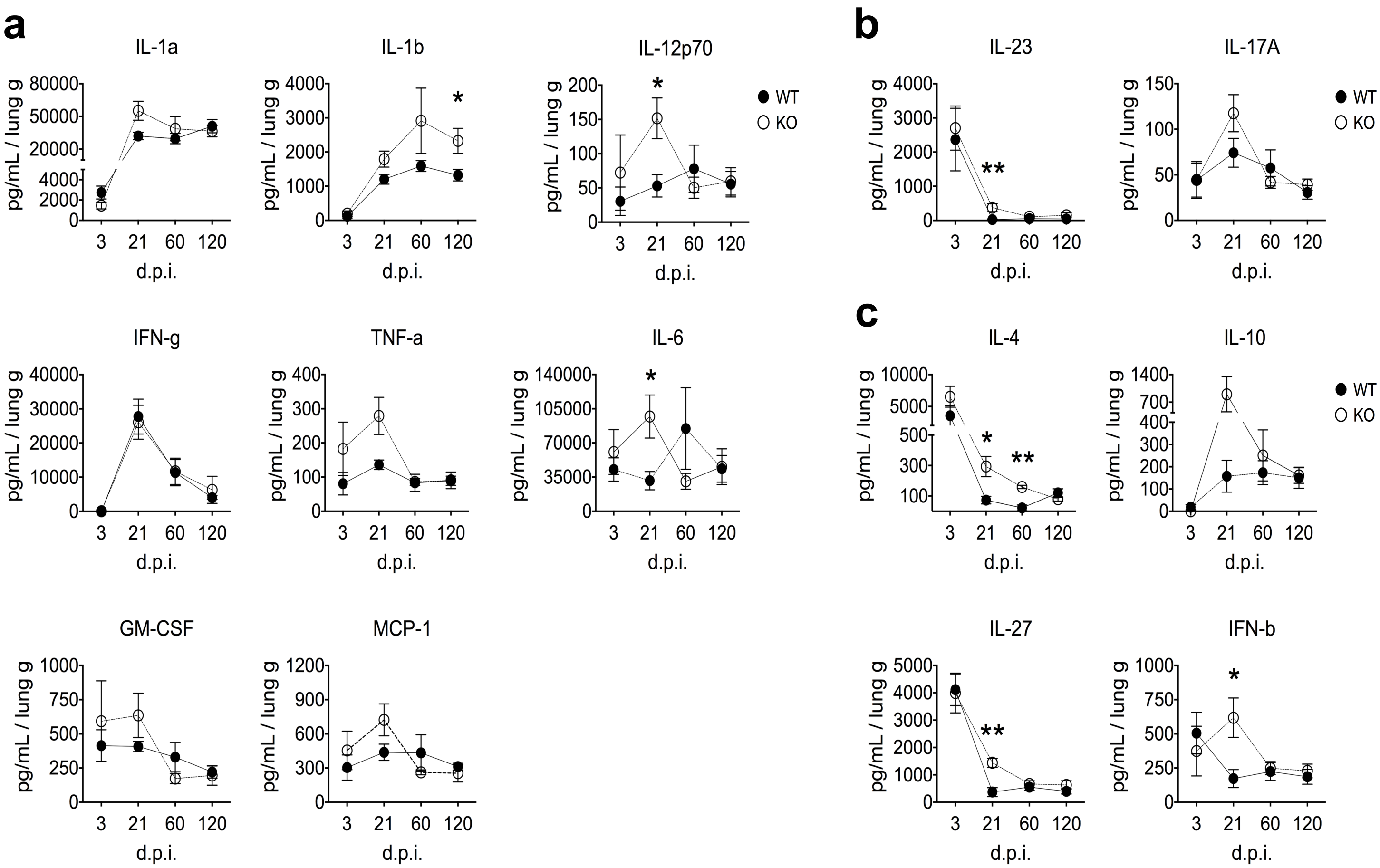

Fig. S3 CD43 deficiency increases the pulmonary production of specific pro-inflammatory, Th17-related, and anti-inflammatory cytokines in BALB/c mice during early TB. Concentration of (**a**) pro-inflammatory cytokines, (**b**) Th17-related cytokines, and (**c**) anti-inflammatory cytokines in the lungs of BALB/c WT and CD43KO mice at 3, 21, 60, and 120 d.p.i. Cytokine concentrations were normalized per lung weight for each mouse. n=5 mice/group (day 3) and n=10 mice/group (days 21, 60, and 120). Statistical analysis: two-tailed Mann-Whitney U test; *p<0.05, **p<0.01. Error bars: SEM.

Fig. S4

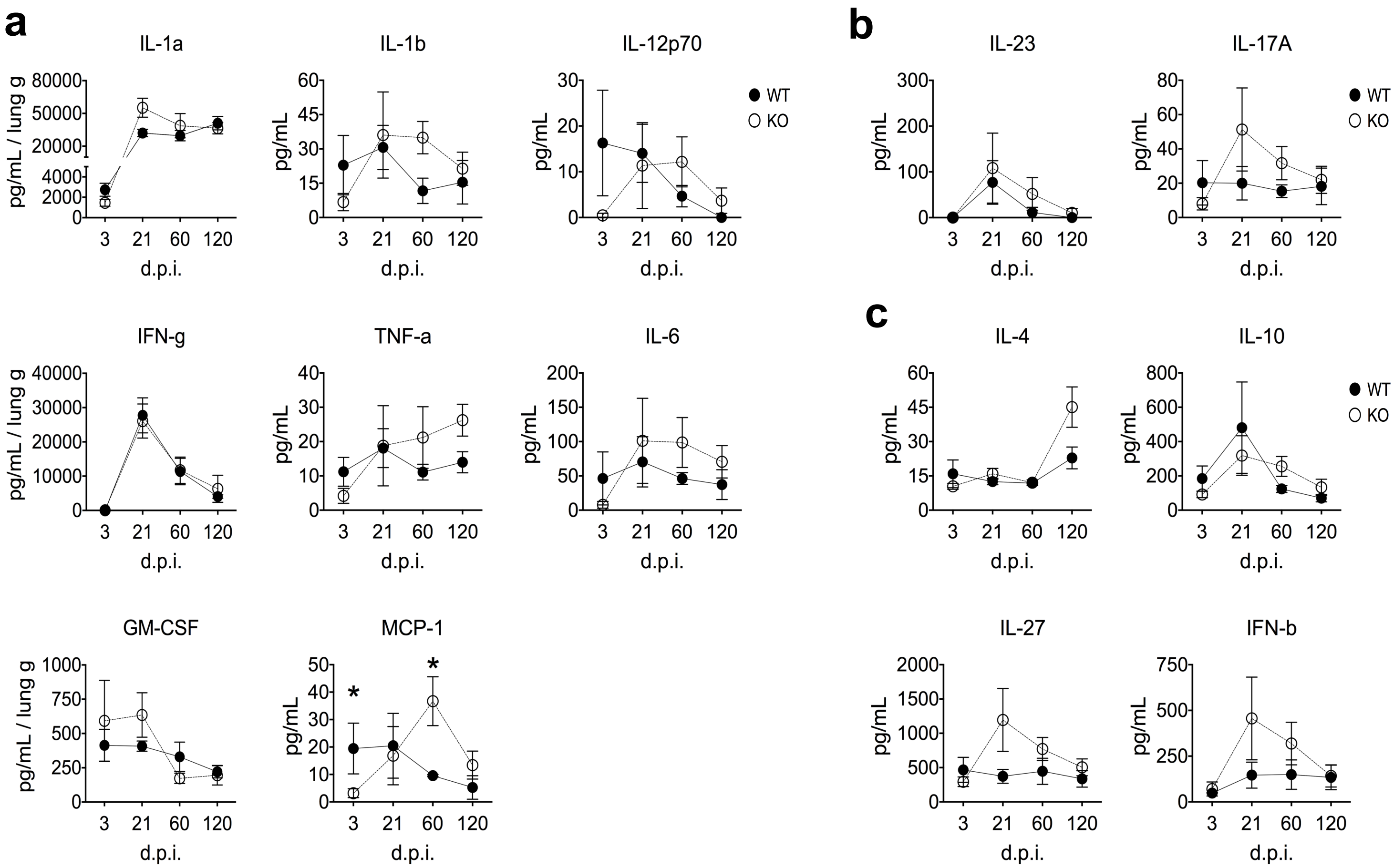

Fig. S4 Serum levels of MCP-1 and IL-1α exhibit distinct, stage-dependent alterations in Mtb-infected BALB/c CD43KO mice. Concentration of pro-inflammatory cytokines (**a**), Th17-related cytokines (**b)**, and anti-inflammatory cytokines (**c**) in the sera of BALB/c WT and CD43KO mice at 3, 21, 60, and 120 d.p.i. n=5 mice/group (day 3) and n=10 mice/group (days 21, 60, and 120). Statistical analysis: two-tailed Mann-Whitney U test; *$p<0.05$, **$p<0.01$. Error bars: SEM.

Fig. S5

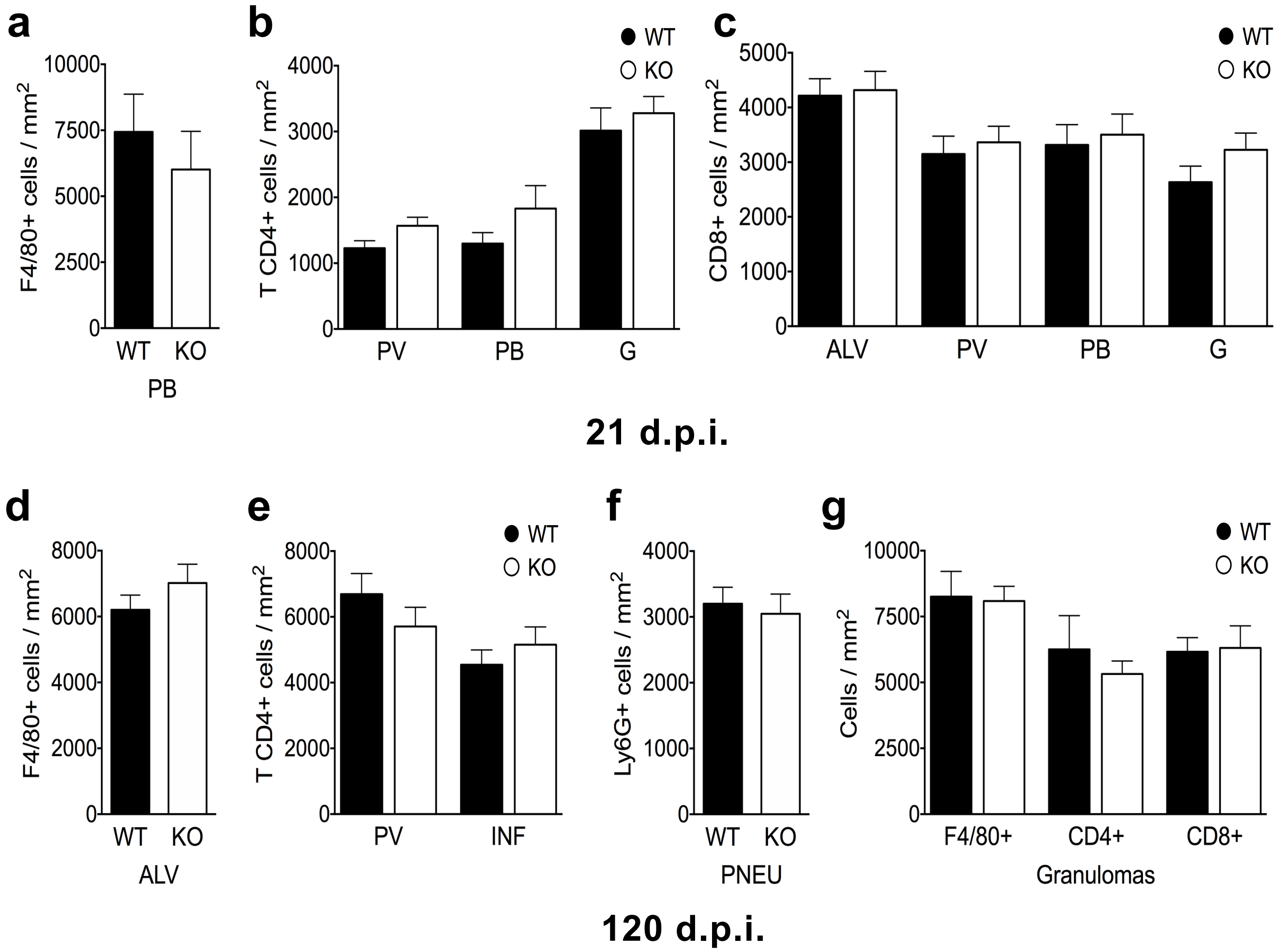

Fig. S5 Pulmonary immune cell populations and compartments showing no significant CD43-dependent changes in BALB/c mice during early and late stages of TB. Quantification (cells/mm²) was performed on immunohistochemistry-stained lung sections from WT and CD43KO mice within manually annotated regions of perivascular (PV) and peribronchial (PB) inflammatory infiltrate, alveolar inflammation (ALV), high cellular infiltration (INF), granulomas (G), and pneumonia (PNEU). **a.** Peribronchial macrophages (F4/80+), 21 d.p.i. **b.** CD4+ T cells in inflammatory infiltrate areas and granulomas, 21 d.p.i. **c.** CD8+ T cells in inflammatory infiltrate areas and granulomas, 21 d.p.i. **d.** Macrophages in alveolar inflammatory areas, 120 d.p.i. **e.** CD4+ T cells in perivascular and highly infiltrated zones, 120 d.p.i. **f.** Neutrophils (Ly6G+) in pneumonic areas, 120 d.p.i. **g.** Macrophages, CD4+ and CD8+ T cells in granulomas, 120 d.p.i. n=10 mice/group. Statistical analysis: two-tailed Mann-Whitney U test. Error bars: SEM.

Fig. S6

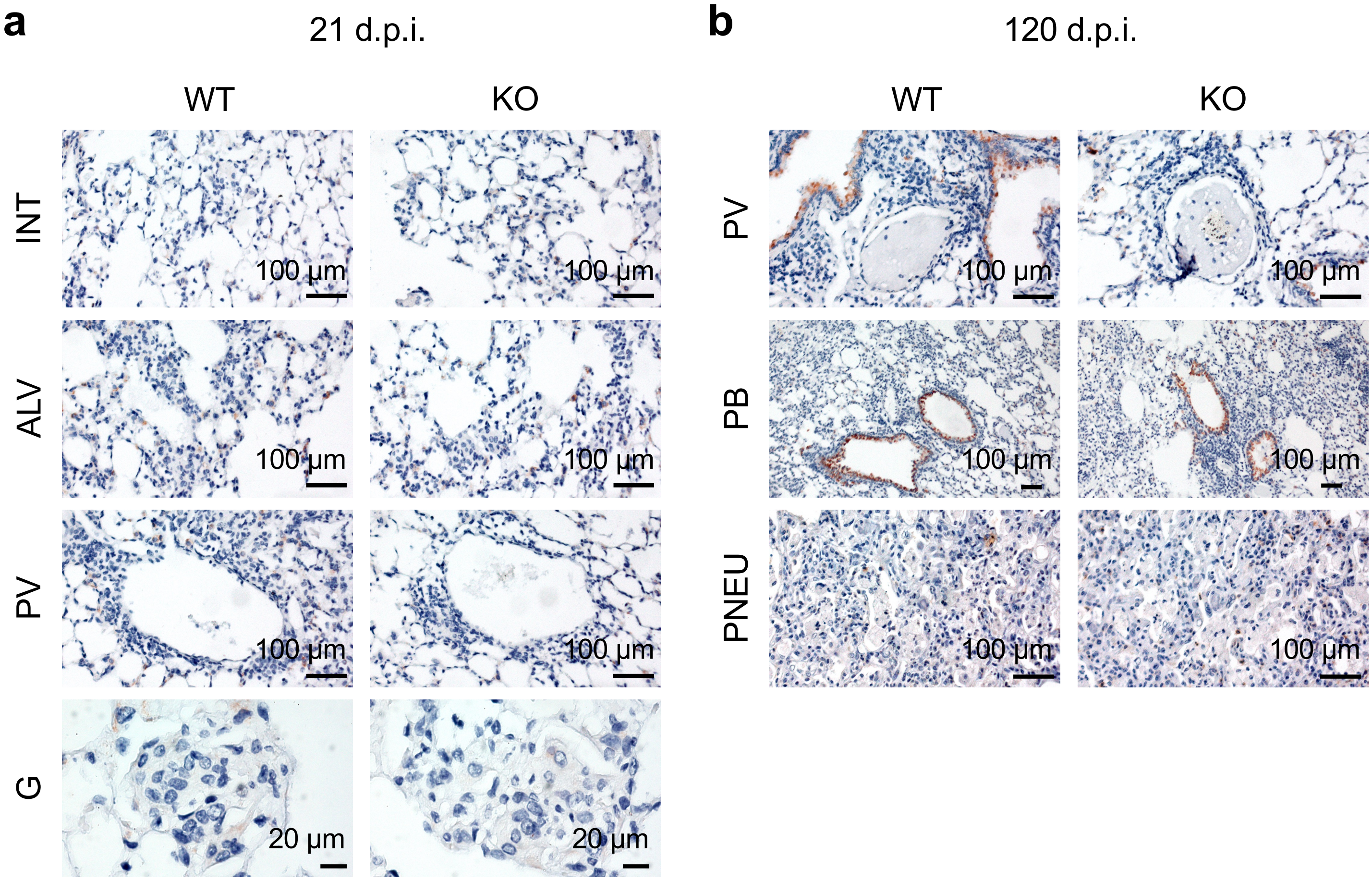

Fig. S6 Negative control images for immunohistochemistry in BALB/c mice. Representative images of lung compartments from WT and CD43KO mice at 21 d.p.i. (**a**) and 120 d.p.i. (**b**). Areas of alveolar-capillary interstitial (INT, 20X), perivascular (PV, 20X), and peribronchial inflammatory infiltrate (PB, 10X), alveolar inflammation (ALV, 20X), pneumonia (PNEU, 20X), and granulomas (G, 63X) are shown.

Fig. S7

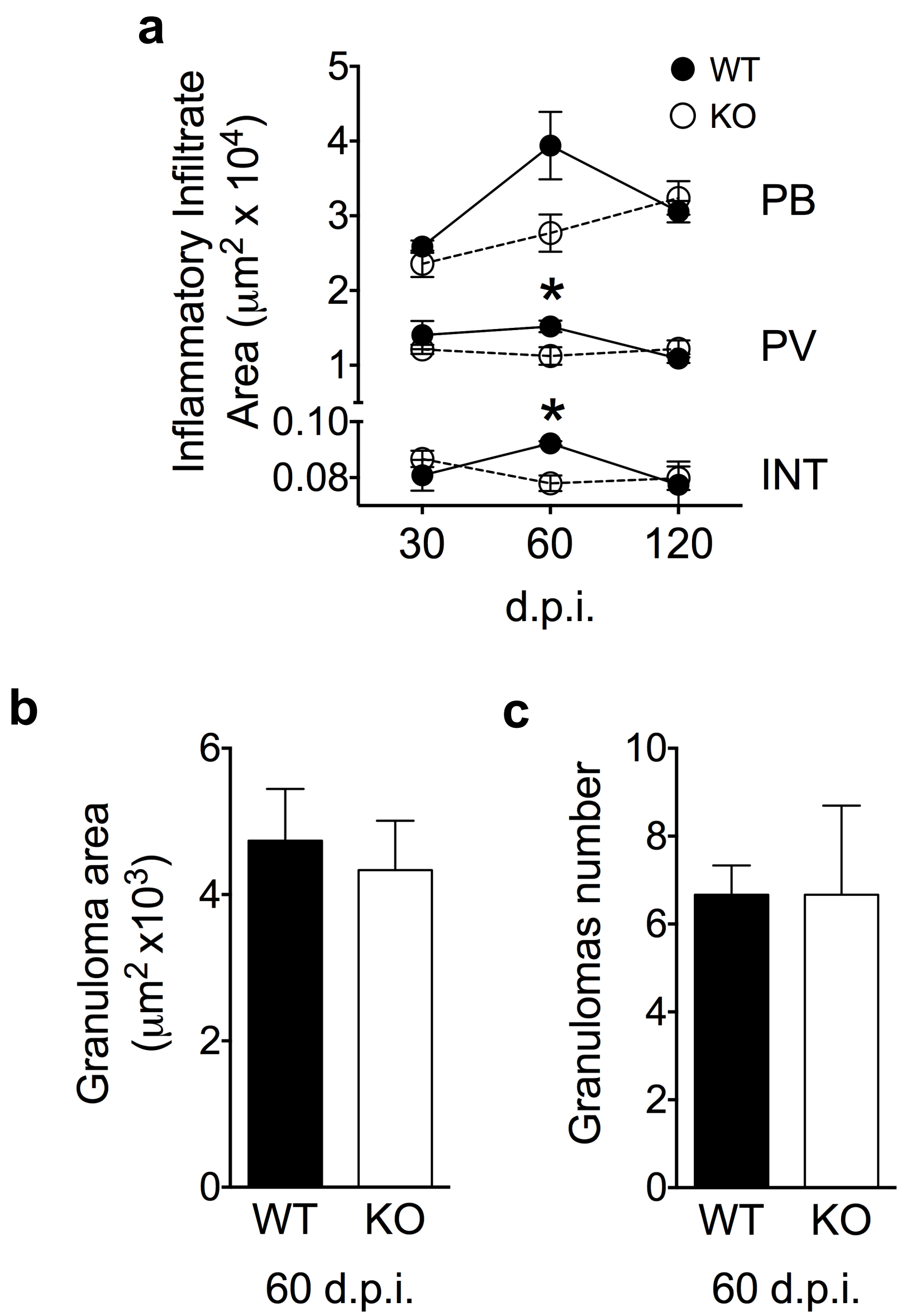

Fig. S7 In C57BL/6 mice, CD43 deficiency reduces inflammatory infiltration without affecting granuloma size or number during late TB. **a.** Area of interstitial (INT), perivascular (PV), and peribronchial (PB) cellular inflammatory infiltrate at 30, 60, and 120 d.p.i., in the lungs of WT and CD43KO mice; morphometric analysis. **b.** Area of single pulmonary granulomas at 60 d.p.i.; morphometric analysis. **c.** Average number of pulmonary granulomas at 60 d.p.i.; morphometric analysis. n=5 mice/group. Statistical analysis: two-tailed Mann-Whitney U test; *$p<0.05$. Error bars: SEM.

Fig. S8

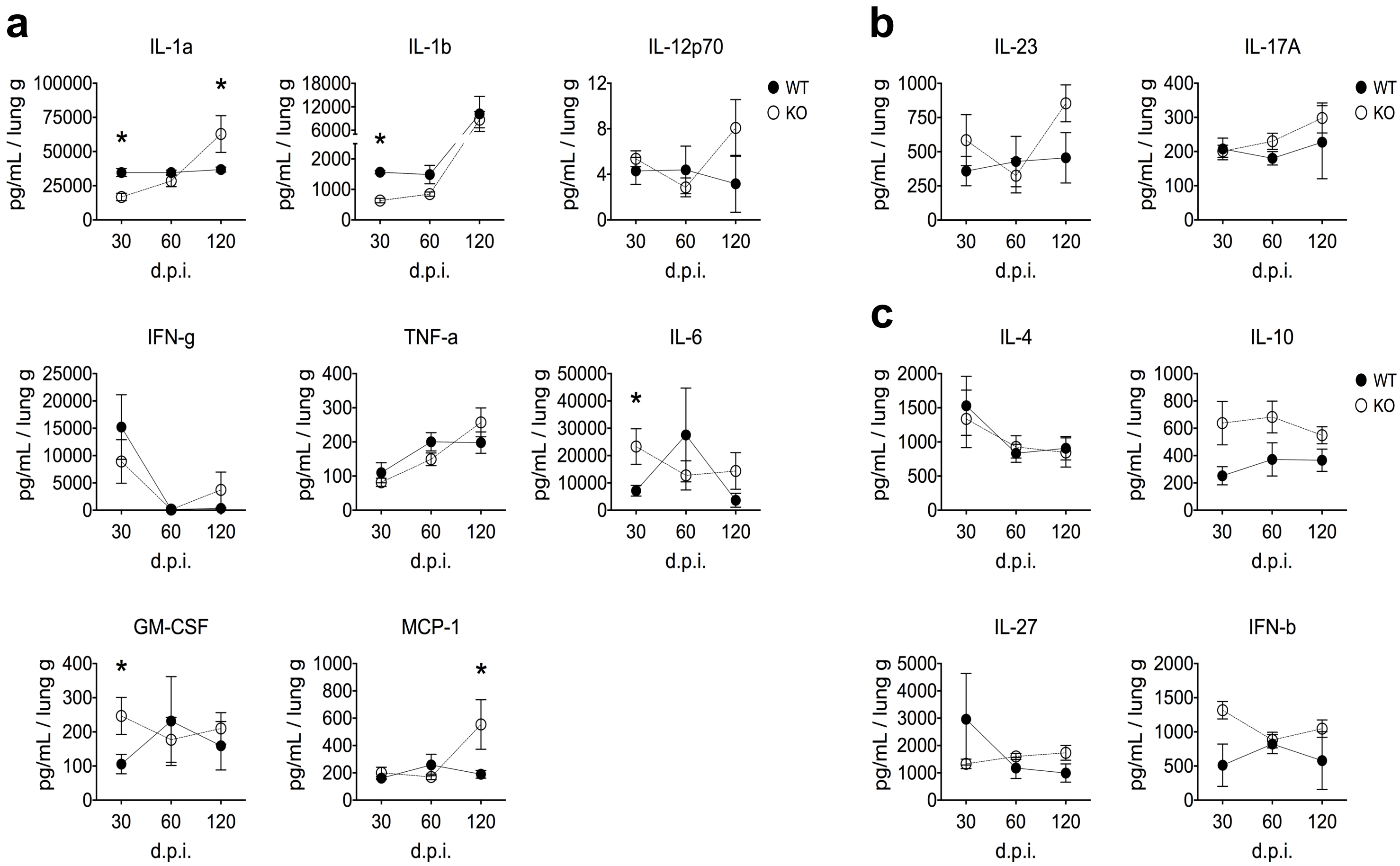

Fig. S8 Pulmonary cytokine profile of C57BL/6 CD43KO mice. Concentration of (**a**) pro-inflammatory cytokines, (**b**) Th17-related cytokines, and (**c**) anti-inflammatory cytokines in the lungs of C57BL/6 WT and CD43KO mice at 30, 60, and 120 d.p.i. Cytokine concentrations were normalized per lung weight for each mouse. n=5 mice/group. Statistical analysis: two-tailed Mann-Whitney U test; *p<0.05. Error bars: SEM.

Fig. S9

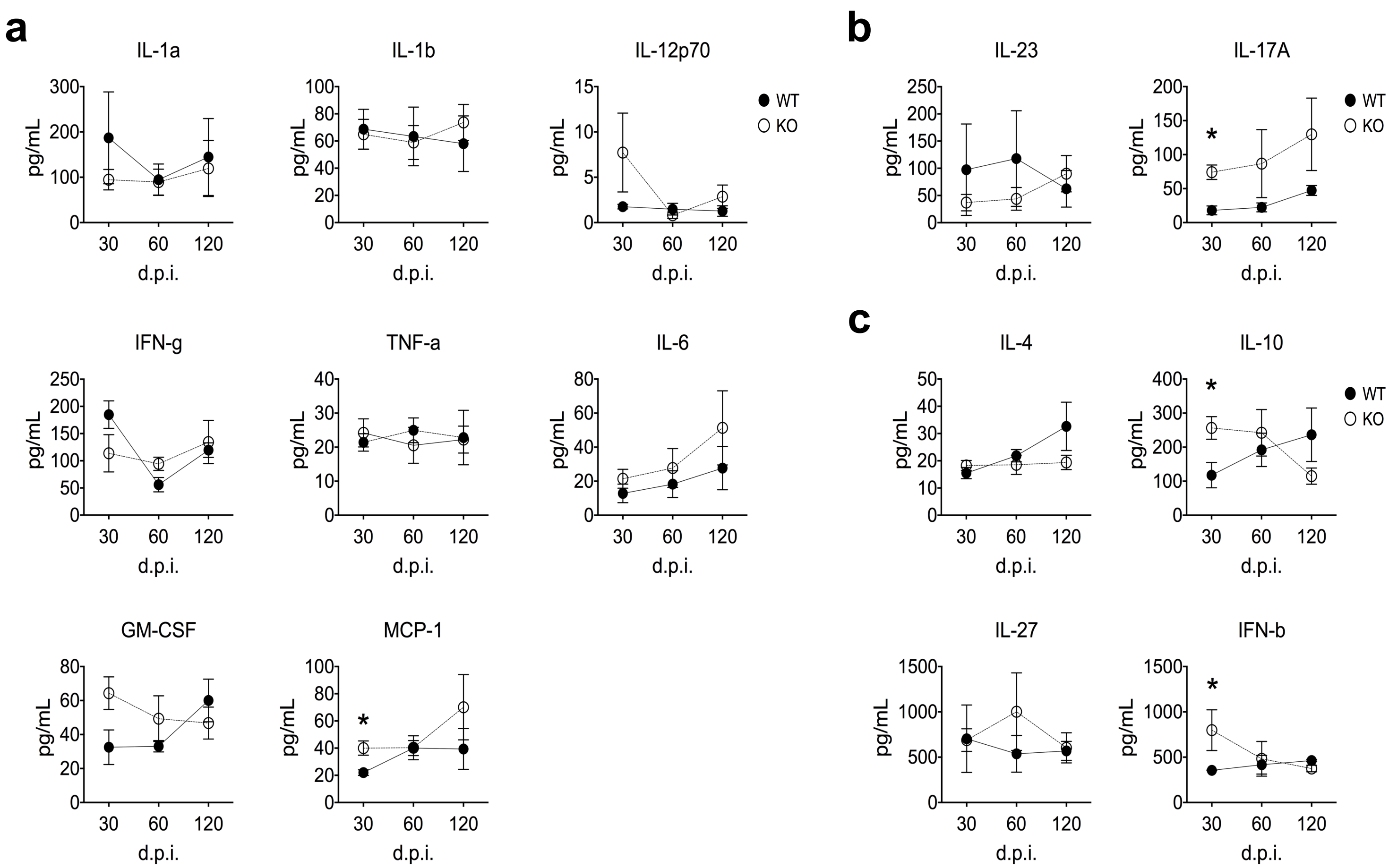

Fig. S9 C57BL/6 CD43KO mice display increased serum levels of MCP-1, IL-17A, IL-10, and IFN-β during early TB. Concentration of (**a**) pro-inflammatory cytokines, (**b**) Th17-related cytokines, and (**c**) anti-inflammatory cytokines in the sera of C57BL/6 WT and CD43KO mice at 30, 60, and 120 d.p.i. (**d.**) Ratio (CD43KO/WT) of circulating cytokine concentrations showing significant differences between groups at 30 d.p.i. n=5 mice/group. Statistical analysis: two-tailed Mann-Whitney U test; *p<0.05. Error bars: SEM.

Fig. S10

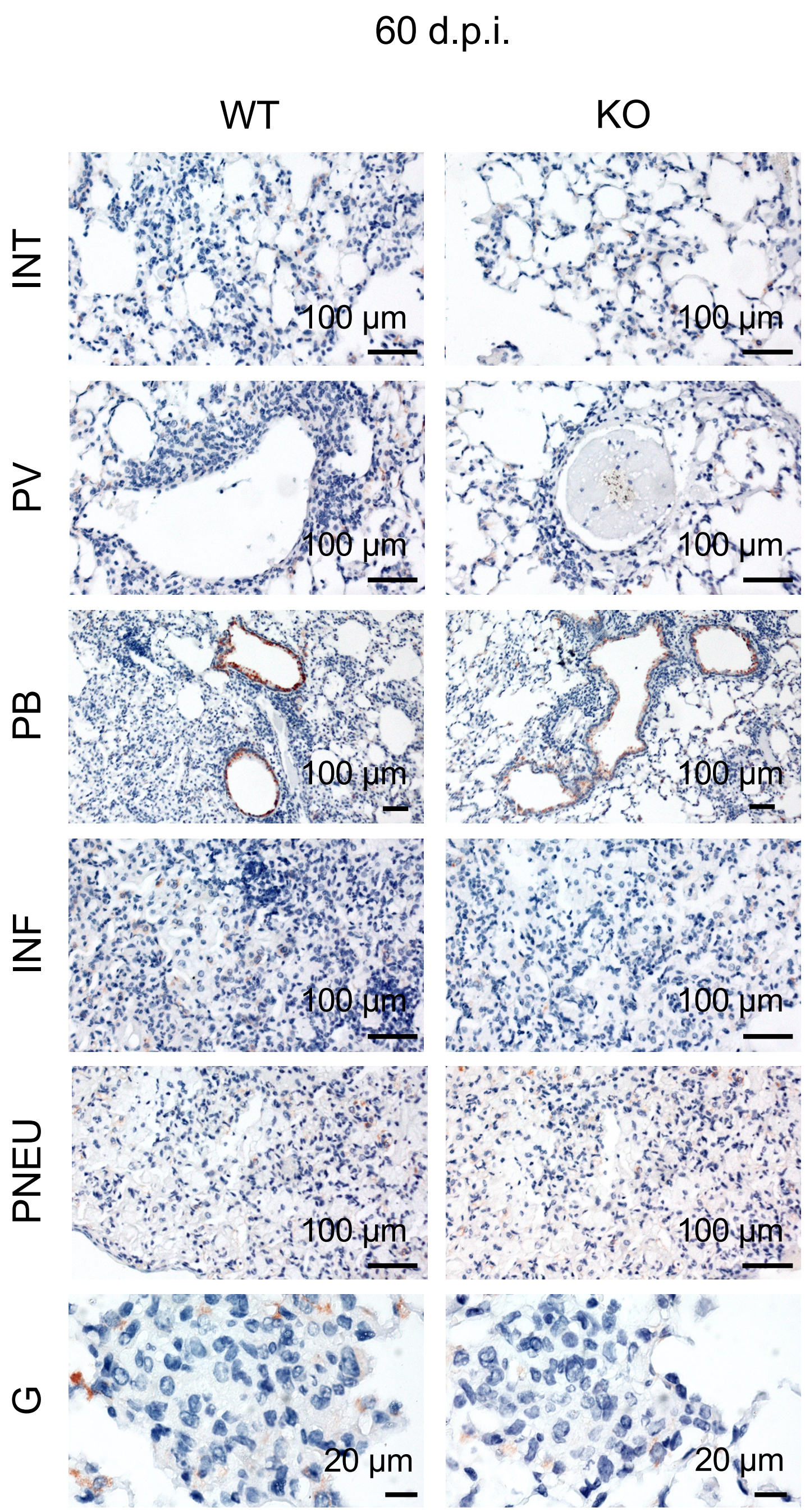

Fig. S10 Negative control images for immunohistochemistry in C57BL/6 mice. Representative images of lung compartments from WT and CD43KO mice at 60 d.p.i. Areas of alveolar-capillary interstitial (INT, 20X), perivascular (PV, 20X), and peribronchial inflammatory infiltrate (PB, 10X), highly infiltrated areas (INF, 20X), pneumonic zones (PNEU, 20X), and granulomas (G, 63X) are shown.

Fig. S11

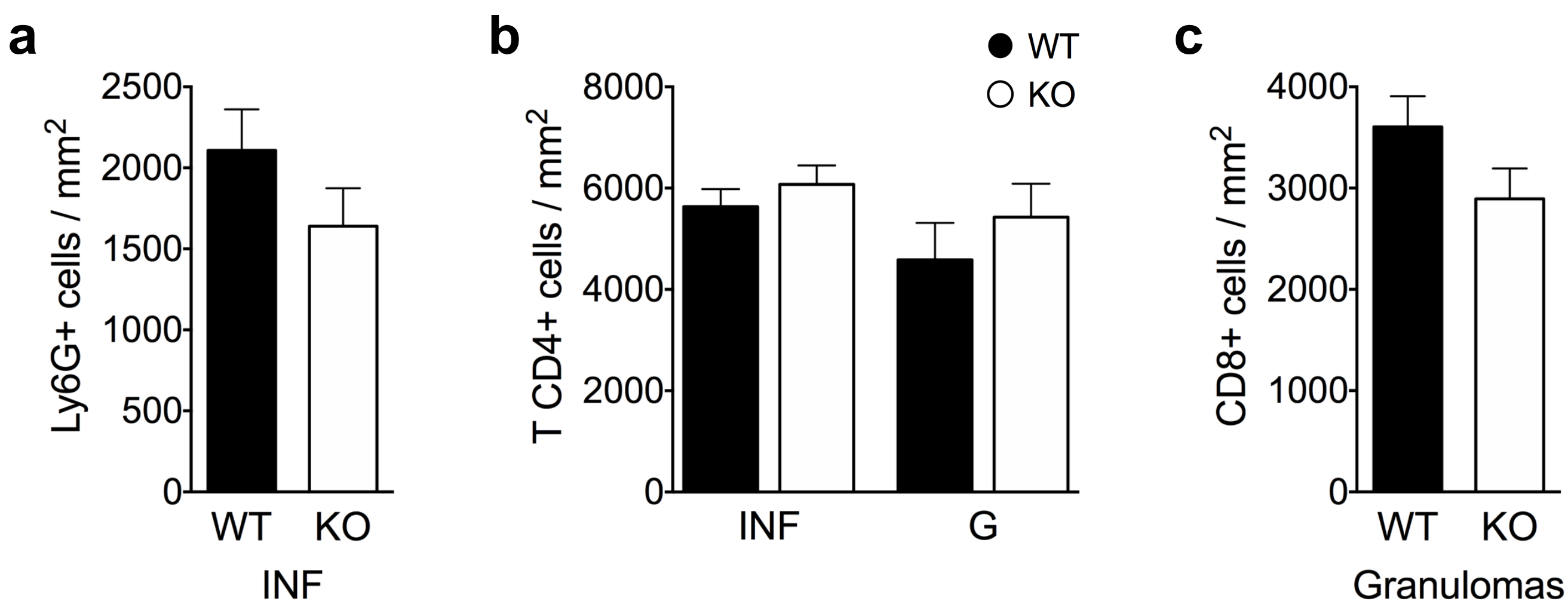

Fig. S11 C57BL/6 WT and CD43KO mice show comparable levels of neutrophils, CD4, and CD8 T cells in highly infiltrated pulmonary zones and granulomas during late TB. Quantification (cells/mm²) was performed on immunohistochemistry-stained manually annotated regions of high cellular infiltration (INF), and granulomas (G) lung sections at 60 d.p.i. **a.** Neutrophils (Ly6G+). **b.** CD4+ T cells (highly infiltrated zones and granulomas). **c.** CD8+ T cells in granulomas. n=5 mice/group. Statistical analysis: two-tailed Mann-Whitney U test. Error bars: SEM.
